# Population Pharmacokinetic Modeling of Glycochenodeoxycholic Acid 3‐O‐Sulfate (GCDCA‐S) as Endogenous Biomarker of OATP1B3 and OAT3 Transporters

**DOI:** 10.1002/cpt.70023

**Published:** 2025-08-11

**Authors:** Yuki Ujihira, Viktor Georgiev, Kayode Ogungbenro, Aleksandra Galetin

**Affiliations:** ^1^ Centre for Applied Pharmacokinetic Research, School of Health Sciences University of Manchester Manchester UK; ^2^ Laboratory for Safety Assessment and ADME, Pharmaceuticals Research Center, Asahi Kasei Pharma Corporation Shizuoka Japan; ^3^ Pharmaceutical Sciences, Roche Innovation Center Welwyn, Roche Pharma Research and Early Development Welwyn UK

## Abstract

Endogenous biomarkers for drug transporters are an emerging tool for the assessment of transporter‐mediated drug–drug interactions (DDI). Glycochenodeoxycholic acid 3‐O‐sulfate (GCDCA‐S) has been proposed as a Tier 2 biomarker of hepatic OATP1B3 and renal OAT3 transporters by the International Transporter Consortium. However, there are currently no mechanistic models developed for this biomarker. This study aimed to characterize the synthesis and elimination of this biomarker through population pharmacokinetic (POP‐PK) modeling of GCDCA‐S plasma and urine data in the presence and absence of OATP1B inhibitor rifampicin and OAT3 inhibitor probenecid. Simultaneous fitting of rifampicin and probenecid interaction data incorporated the inhibitory effect of rifampicin on GCDCA‐S hepatic clearance (CL_h_) and probenecid inhibitory effect on both renal (CL_R_) and hepatic (CL_h_) clearance parameters, assuming no effect on the synthesis of GCDCA‐S. The POP‐PK model successfully described the observed data for GCDCA‐S, with reasonable standard errors (<40%) for population parameter estimates. The results indicated biliary excretion as the primary route of elimination for GCDCA‐S (~ 95%). The GCDCA‐S model was successfully verified against four independent datasets on plasma baseline and interaction after rifampicin administration. Power calculations confirmed the sensitivity of GCDCA‐S for identifying weak to strong OATP1B3 and OAT3 inhibitors using plasma AUC and renal clearance as metrics, respectively. This study provides further validation of GCDCA‐S as an endogenous biomarker of OATP1B3 and OAT3 transporters and offers a valuable resource for optimizing the design of prospective OATP1B3‐ and OAT3‐mediated DDI studies in early‐phase clinical trials.

**Study Highlights**

**WHAT IS THE CURRENT KNOWLEDGE ON THE TOPIC?**

Glycochenodeoxycholic acid 3‐O‐sulfate (GCDCA‐S) has been identified as a biomarker of hepatic OATP1B3 and renal OAT3 transporters. Plasma concentrations of GCDCA‐S increase following the administration of OATP1B inhibitors, while renal clearance decreases after the administration of OAT3 inhibitors.

**WHAT QUESTION DID THIS STUDY ADDRESS?**

Population pharmacokinetic models were developed to support qualification of GCDCA‐S as an endogenous biomarker of OATP1B3 and OAT3 transporters. Power calculations were performed to assess the sensitivity of GCDCA‐S and to guide the optimal design of clinical DDI studies.

**WHAT DOES THIS STUDY ADD TO OUR KNOWLEDGE?**

This study is the first to successfully quantify the synthesis rate and systemic elimination pathways of GCDCA‐S. The results indicated biliary excretion as the primary route of elimination for GCDCA‐S (~95%). Power calculation confirmed the sensitivity of GCDCA‐S in identifying weak to strong OATP1B3 and OAT3 inhibitors.

**HOW MIGHT THIS CHANGE CLINICAL PHARMACOLOGY OR TRANSLATIONAL SCIENCE?**

Modeling and simulation support the utility of GCDCA‐S as a selective endogenous biomarker for detecting OATP1B3‐ and OAT3‐mediated DDIs.

Monitoring of endogenous biomarkers of clinically relevant transporters in the early phases of clinical development has gained a lot of interest as an alternative approach for assessing transporter‐mediated drug–drug interactions (DDI) of new molecular entities.^1^, ^2^, ^3^, ^4^, ^5^ This approach enables assessment of *in vivo* DDI risks without the need for administration of probe drugs. Use of biomarker interaction data to estimate *in vivo* Ki enables the refinement of inhibitor PBPK models, thereby facilitating the design and optimization of subsequent clinical DDI studies.^6^, ^7^, ^8^, ^9^, ^10^, ^11^, ^12^, ^13^ Recently, a tier classification system for transporter biomarkers has been proposed by the International Transporter Consortium (ITC).^1^ The classification is based on the selectivity and sensitivity of the biomarker for its target, as well as the availability of clinical DDI data and validated mechanistic models for these biomarkers.^1^ Coproporphyrin‐I (CP‐I; OATP1B biomarker) is the only fully validated endogenous biomarker to date (Tier 1 biomarker). Monitoring changes in CP‐I maximum plasma concentration (*C*_max_) or area under the concentration‐time curve (AUC) in the presence of a transporter inhibitor in single or multiple ascending dose studies is recommended to guide the decision‐making with respect to OATP1B DDI study conduct.^1^, ^3^ Glycochenodeoxycholic acid 3‐O‐sulfate (GCDCA‐S) has been identified as a biomarker for OATP1B3 and OAT3 transporters.^1^, ^12^ Although monitoring of its concentrations alongside Tier 1 biomarkers is advised, it has not yet reached the level of maturity to inform decision‐making and hence it is currently classified as a Tier 2 biomarker.^1^ GCDCA‐S plasma concentrations have been reported to increase after the administration of OATP1B inhibitors,^14^, ^15^, ^16^, ^17^, ^18^, ^19^, ^20^, ^21^ with the observed AUCR (AUC_+inhibitor_/AUC_control_) greater than that of CP‐I, indicating greater sensitivity. Furthermore, GCDCA‐S has been reported as the most sensitive OAT3 biomarker (based on the changes in its renal clearance) in clinical studies evaluating multiple OAT1/3 biomarkers (e.g., 4‐pyridoxic acid (PDA), homovanillic acid).^7^, ^22^, ^23^ Despite these promising findings, there are currently no mechanistic models developed for this biomarker, and its synthesis rate and relative contribution of the renal and biliary routes of elimination remain unclear. The lack of modeling efforts for GCDCA‐S contributes significantly to its current status as a Tier 2 biomarker.^1^

Bile acids have a complex biosynthesis pathway (**Figure** **1**). The primary bile acids, including chenocholic acid (CA) and chenodeoxycholic acid (CDCA) are products of cholesterol catabolism, approximately 90% of which takes place in hepatocytes.^24^, ^25^ Primary bile acids become more hydrophilic and are secreted into bile through sulfation or conjugation with taurine or glycine.^24^, ^25^, ^29^ Sulfation via sulfotransferase 2A1 (SULT2A1) represents a major elimination pathway for bile acids in humans.^29^, ^30^, ^31^, ^32^ Bile acids sulfates, including GCDCA‐S, are more water‐soluble and more readily excreted in feces and urine compared to their non‐sulfated form.^29^, ^30^, ^31^, ^32^ While non‐sulfated bile acids (e.g., CA, CDCA, glycochenodeoxycholic acid (GCDCA)) are known to undergo enterohepatic circulation, bile acid sulfates are expected to undergo limited enterohepatic recirculation because of their limited absorption in the intestine.^29^, ^30^, ^31^, ^32^

**Figure 1:**
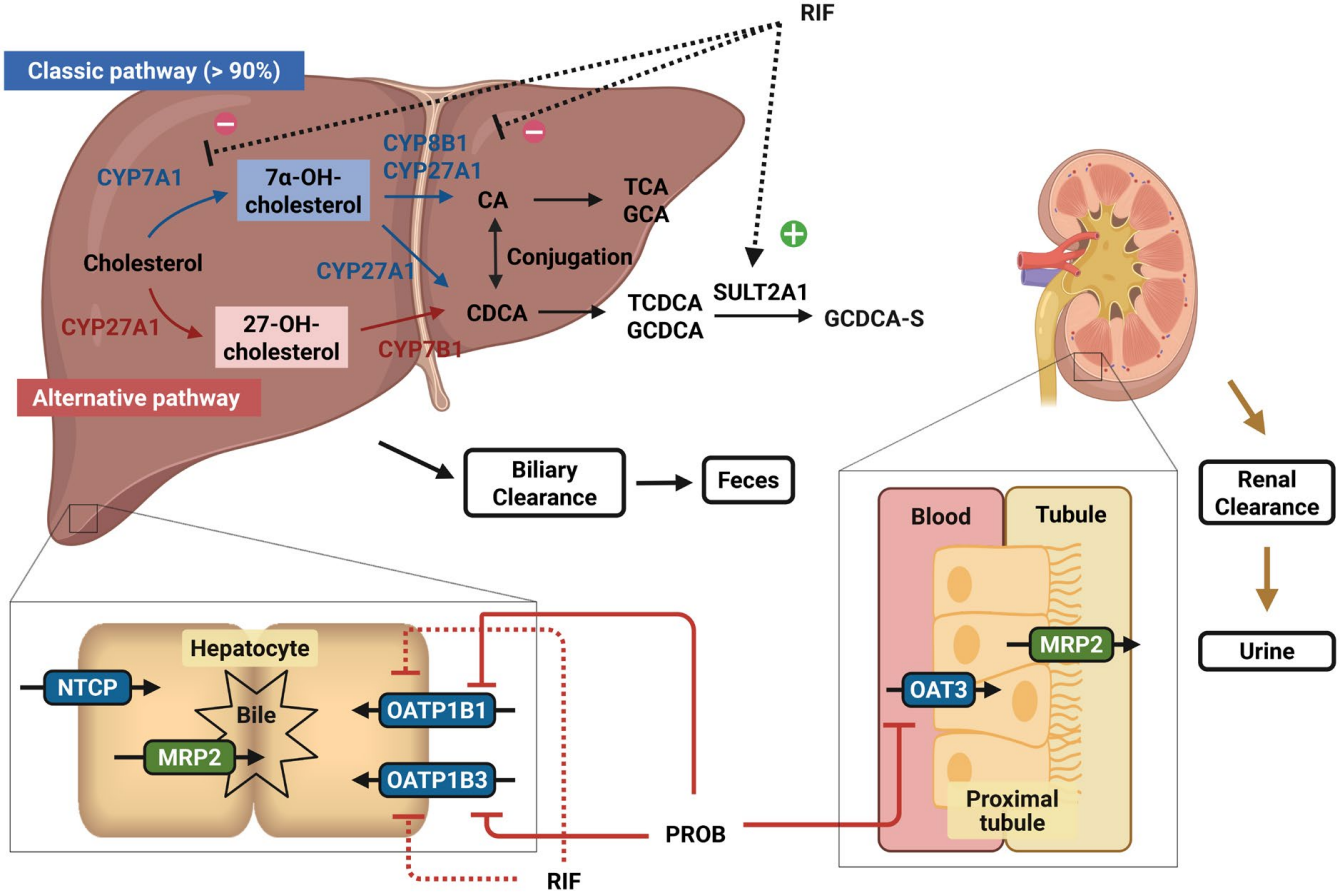
Synthetic and elimination pathways of GCDCA‐S. Bile acid synthesis proceeds via two distinct pathways: the classic pathway, which utilizes CYP7A1, and the alternative pathway, which is mediated by CYP27A1.^24^, ^25^ These pathways serve as the primary route for bile acid synthesis, accounting for more than 90% of total bile acid production in humans.^24^, ^25^ Many bile acids (e.g., CDCD, GCDCA), some of which are precursors of GCDCA‐S, have been reported as substrates of OATP1B transporters.^14^, ^15^, ^26^, ^27^ GCDCA‐S is eliminated via biliary and renal excretion. In the liver, OATP1B1/3 and NTCP transporters actively uptake GCDCA‐S from the portal blood in hepatocytes, after which it is secreted into the bile by MRP2 transporters.^2^, ^14^, ^28^ In the kidney, OAT3 transporters actively uptake GCDCA‐S from the renal blood into the proximal tubular cell.^2^, ^22^, ^23^ This process is followed by the apical efflux of GCDCA‐S from the proximal tubular cell into the tubular lumen via MRP2.^22^ CA, chenocholic acid; CDCA, chenodeoxycholic acid; CYP7A1, cholesterol 7α‐hydroxylase; CYP27A1, mitochondrial sterol 27‐hydroxylase; GCDCA, glycochenodeoxycholic acid; GCDCA‐S, glycochenodeoxycholic acid 3‐O‐sulfate; MRP2, multidrug resistance‐associated protein 2; NTCP, sodium taurocholate co‐transporting polypeptide; OAT, organic anion transporters; OATP, organic anion‐transporting polypeptides; SULT2A1, enzyme sulfotransferase 2A1; TCA, taurocholic acid; TCDCA, taurochenodeoxycholic acid. This figure was created with BioRender.com.

Modeling and simulation are increasingly being utilized as tools to gain mechanistic insights into the properties of transporter biomarkers.^1^, ^3^, ^4^, ^6^, ^7^, ^9^, ^10^, ^11^, ^12^ Early modeling efforts for CP‐I and PDA (a biomarker for OAT1/3 transporters) focused on characterizing their synthesis rate and delineating the relative contribution of their elimination pathways through population pharmacokinetic (POP‐PK) modeling.^6^, ^7^ Based on this information, physiologically based pharmacokinetic (PBPK) models have subsequently been developed and used to estimate the *in vivo* transporter inhibition potential of investigational drugs.^8^, ^9^, ^10^, ^13^, ^33^, ^34^

To evaluate the utility of GCDCA‐S as an endogenous biomarker for OATP1B3‐ and OAT3‐mediated DDIs, this study aimed to: (1) characterize the synthesis and elimination of this biomarker through POP‐PK modeling of GCDCA‐S plasma and urine data; (2) perform simulations to assess the sensitivity of GCDCA‐S to identify weak and moderate OATP1B3 and OAT3 inhibitors; (3) perform power calculations to inform the design of prospective clinical DDI studies where GCDCA‐S may be monitored.

## METHODS

### Clinical data used for model development

Individual plasma and urine data of GCDCA‐S in healthy subjects were obtained from three clinical studies. Tatosian *et al*.^18^ (study #1) consisted of two occasions: on both occasions, a microdose probe drug cocktail for multiple clinically relevant transporters was administered, with a single oral dose of 600 mg rifampicin co‐administered on the second occasion (*n* = 6; 3 males, 3 females). Willemin *et al*.^22^ (study #2) was a two‐phase, cross‐over study with a 21‐day washout period between the two phases. GCDCA‐S plasma concentrations and amount in urine were measured both in the absence and presence of probenecid, with patients receiving 500 mg approximately every 6 hours for 7 days while subjects were also receiving the Johnson and Johnson compound (*n* = 6; 6 females). Study #3 consists of unpublished data from the same study design as reported by Willemin *et al*. (*n* = 12; 12 males).^22^ Demographic information, sample collection, and dosing conditions are summarized in **Table** **1**.

**Table 1:** Clinical data used for GCDCA‐S population PK model development.

	Study #1 (Tatosian *et al*.^18^)	Study #2 (Willemin *et al*.^22^)	Study #3 (unpublished data)
Ethnicity	White	White	White
Sex (No. subjects)	Male (3), Female (3)	Female (6)	Male (12)
Age range (year)	50–71	48–58	20–55
Duration of sample collection (h)	24	168	24
Food intake	Unknown^a^	Unknown^a^	
Inhibitor	RIF	PROB	
No. occasions; treatment	Occ1; Cocktail of probe drugs Occ2; Cocktail of probe drugs + RIF	Occ1; Johnson and Johnson NCE^b^ Occ2; Johnson and Johnson NCE^b^ + PROB	
Inhibitor Dosing	Single dose of 600 mg	500 mg oral dose at 6 pm and 11 pm on day 0, followed by the same dose at 7 am, 1 pm, 6 pm, and 11 pm on days 1 to 7^c^	
No. GCDCA‐S plasma samples	Occ1; 48, Occ2; 48	Occ1; 62, Occ2; 66	Occ1; 108, Occ2; 98
No. GCDCA‐S urine samples	0	Occ1; 54, Occ2; 53	Occ1; 36, Occ2; 32
No. inhibitor plasma samples	Occ2; 51	Occ2; 36	Occ2; 66
C_baseline_ d (nM)	86	64	87

C_baseline_, GCDCA‐S plasma baseline concentration in the absence of inhibitors; GCDCA‐S, glycochenodeoxycholic acid 3‐O‐sulfate; NCE, new chemical entity; PROB, probenecid; RIF, rifampicin.

^a^ Information regarding food intake was not reported in the original publication.^18^, ^22^

^b^ Johnson and Johnson NCE did not inhibit the transporters targeted in this study (OATP1B3 and OAT3).

^c^ Plasma sample collection begins at day 1 of the study.

^d^ Average concentration from 0 to 24 hours.

### POP‐PK model development and assessment

**Figure 2:**
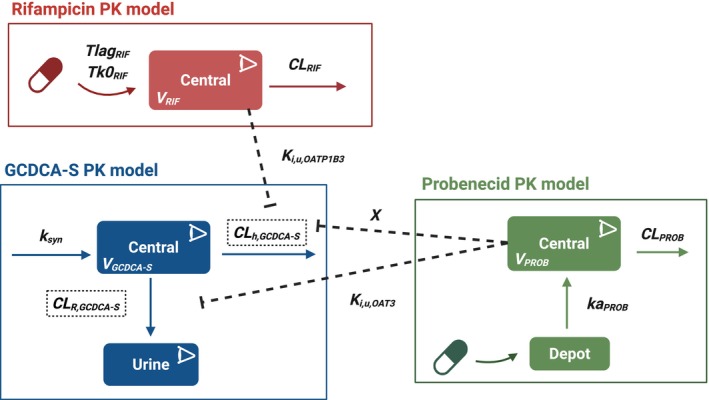
Schematic diagram of population PK models for rifampicin, probenecid, and GCDCA‐S. CL_h_, hepatobiliary clearance; CL_R_, renal clearance; X, fold reduction in CL_h_; GCDCA‐S, glycochenodeoxycholic acid 3‐O‐sulfate; *k*_*a*_, absorption rate constant; *k*_syn_, synthesis rate; *K*_*i*,u,OAT3_, probenecid unbound OAT3 inhibitory constant; *K*_*i*,u,OATP1B3_, rifampicin unbound OATP1B3 inhibitory constant; OAT, organic anion transporters; OATP, organic anion‐transporting polypeptides; PROB, probenecid; RIF, rifampicin; Tk0_RIF_, duration of zero‐order absorption; Tlag_RIF_, lag time. This figure was created with BioRender.com.

Population PK models for rifampicin, probenecid, and GCDCA‐S were developed in Monolix 2024R2 (Lixoft, France) (**Figure** **2**). Models were evaluated using visual predictive check (VPC) plots, goodness‐of‐fit (GOF) plots, parameter uncertainty, and changes in objective function. Model visualization and simulations were conducted in MATLAB 2024a (MathWorks, Natick, MA, USA) and Graph Pad Prism 10.2.2 (GraphPad software, La Jolla, CA, USA).

### Structural models for rifampicin, probenecid, and GCDCA‐S

The structural model for rifampicin was based on our previous report,^33^ and represents a one‐compartment model with zero‐order absorption and linear elimination. In the case of probenecid, the structural model was adopted from Ahmad *et al*.^7^ and represents a one‐compartment model with first‐order absorption and linear elimination. The structural model for GCDCA‐S was based on our previously published biomarker POP‐PK models.^6^, ^7^ It represents a turnover model with zero‐order synthesis and first‐order hepatobiliary and renal clearances, necessary to capture the synthesis rate of GCDCA‐S and the relative contributions of the hepatobiliary and renal elimination routes (Eqs (1), (2), (3), (4)).

GCDCA‐S plasma concentrations fluctuate, despite being reported to have less pronounced diurnal variations in plasma baseline compared with its precursor GCDCA.^15^, ^19^ Different functions for evaluating diurnal fluctuation (e.g., Bateman, Power function) were investigated and applied to the synthesis rate in the GCDCA‐S model. The inhibitory effect of rifampicin on the hepatic uptake clearance of GCDCA‐S via OATP1B3 was reflected in GCDCA‐S hepatic clearance (CL_h_). It was assumed that rifampicin has no effect on the CL_R_ of GCDCA‐S, in agreement with the available clinical DDI data.^15^ The inhibitory effect of probenecid on the active secretory clearance of GCDCA‐S via OAT3 was accounted for in GCDCA‐S renal clearance (CL_R_). *In vitro* studies reported that probenecid weakly inhibits OATP1B transporters.^35^ The effect of probenecid on OATP1B3 was estimated as the fold decrease in GCDCA‐S CL_h_ (defined as X) in the presence of probenecid. Due to incomplete inhibition and lack of dose range data, the estimation of the OATP1B3 inhibition constant for probenecid was considered unreliable. Both rifampicin and probenecid models assumed that neither inhibitor had an effect on the synthesis of GCDCA‐S. Simultaneous fittings of the clinical data in the absence and presence of rifampicin and probenecid were performed. Equations 1 and 2 describe changes in GCDCA‐S plasma concentration in the absence and presence of inhibitors, whereas Eqs 3 and 4 show the corresponding changes in the amount of GCDCA‐S in urine, respectively.

(1) $$\frac{{dC}_{\text{GCDCA}-S}}{dt}=\left[k_{\mathrm{syn}}-\left(C_{\text{GCDCA}-S}\times CL_{h}+C_{\text{GCDCA}-S}\times CL_{R}\right)\right]\times \frac{1}{\mathrm{Vc}}$$

(2)

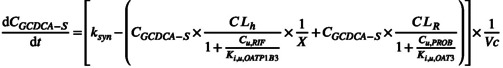

(3) $$\frac{{dA}_{\text{GCDCA}-S}}{dt}=C_{\text{GCDCA}-S}\times CL_{R}$$

(4) $$\frac{{dA}_{\text{GCDCA}-S}}{dt}=C_{\text{GCDCA}-S}\times \frac{CL_{R}}{1+\frac{C_{u,\text{PROB}}}{\mathrm{Ki}\;u\;\mathrm{OAT}3}}$$

where *C*_*GCDCA–S*_ (μM) is GCDCA‐S plasma concentration, *A*_*GCDCA–S*_ (μmol) is GCDCA‐S amount in urine, *k*_*syn*_ (μmol/h) is the zero‐order GCDCA‐S synthesis rate, *CL*_*h*_ (L/h) is hepatic clearance of GCDCA‐S, *CL*_*R*_ (L/h) is renal clearance of GCDCA‐S, *V*_*C*_ (L) is the volume of distribution of GCDCA‐S, *K*_*i,u,OATP1B3*_ (μM) is unbound OATP1B3 inhibition constant for rifampicin, *C*_*u,RIF*_ (μM) is unbound plasma concentration for rifampicin (linked to RIF model), *K*_*i,u,OAT3*_ (μM) is unbound OAT3 inhibition constant for probenecid, *C*_*u,PROB*_ (μM) is unbound plasma concentration for probenecid (linked to PROB model), and *X* is a fold reduction in hepatic clearance of GCDCA‐S in the presence of probenecid (*X* = 1 in the absence of probenecid).

### Statistical models

The models for rifampicin, probenecid, and GCDCA‐S incorporated both inter‐individual variability for a number of model parameters (assuming log‐normal distribution) and residual unexplained variability (using a combination of proportional and additive error models).

### Covariate analysis – Sex effect

Serum concentrations of primary bile acids (CA, CDCA) and fasting plasma concentrations of GCDCA‐S have been shown to be higher in males than in females.^24^, ^36^, ^37^, ^38^ Therefore, sex was explored as a covariate and incorporated into the synthesis rate of GCDCA‐S (details in the **Supplementary Material** **S1**).

### GCDCA‐S model verification

Model verification was performed using four independent clinical datasets with reported GCDCA‐S plasma concentrations. Details of the clinical studies are in **Table** **S1**. These datasets included two studies conducted in Caucasians, Orozco *et al*. (baseline data)^36^ and Robbins *et al*. (baseline and rifampicin DDI data),^17^ as well as two studies conducted in Japanese individuals^15^, ^16^ where the change in plasma GCDCA‐S exposure was evaluated at different rifampicin doses. GCDCA‐S plasma concentrations were digitized using Web Plot Digitizer (https://automeris.io/WebPlotDigitizer/). Simulations were conducted in agreement with the reported study designs. The median (50^th^ percentile) of the predicted concentrations, along with the 90% prediction interval (5^th^ and 95^th^ percentiles), was plotted and compared against the observed data.

### Power calculation

To evaluate the utility of GCDCA‐S in identifying weak and moderate OATP1B3 and OAT3 inhibitors, GCDCA‐S plasma AUC and CL_R_ were simulated across different exposure levels and inhibitor potencies (I/Ki) ratios relative to that of rifampicin and probenecid using the same approach as in our previous reports^6^, ^7^ (details are provided in the **Supplementary Material** **S1**). The plasma concentration, amount in urine, and renal clearance of GCDCA‐S were simulated using the rifampicin, probenecid, and GCDCA‐S models in MATLAB, based on the study designs reported by Tatosian *et al*.^18^ and Willemin *et al*.^22^ Power curves were calculated for each modified I/Ki ratio by performing a one‐sample paired t‐test on the log‐transformed AUC (for detecting OATP1B3‐mediated DDIs) and CL_R_ (for detecting OAT3‐mediated DDIs) ratios after simulations with predefined sample sizes. For each sample size (ranging from 5 to 30), simulations and statistical tests were repeated 1,000 times. The statistical power of the test was determined as the proportion of simulations in which the null hypothesis was rejected at a significance level of 0.01 and 0.05.

### Potential effect of transporter inhibitor on GCDCA‐S synthesis rate

No information on the effect of probenecid on GCDCA‐S *k*_syn_ was available in the literature. Rifampicin has been reported to inhibit CYP7A1 and CYP8B1,^14^, ^15^, ^39^, ^40^ while it has also been suggested to induce SULT2A1, the final step in the GCDCA‐S synthesis (**Figure** **1**).^41^, ^42^ Plasma concentrations of many bile acids (e.g., GCDCA), which are precursors of GCDCA‐S, have been reported to increase following rifampicin administration.^14^, ^15^, ^26^, ^27^ Due to insufficient data, these effects could not be incorporated into the GCDCA‐S model. The potential effect of rifampicin and probenecid on GCDCA‐S synthesis, in addition to their respective inhibitory effects on OATP1B3‐ and OAT3‐mediated transport, was investigated by simulation (details in the **Supplementary Material** **S1**).

## RESULTS

### Analysis of individual clinical GCDCA‐S data

The observed data used for POP‐PK model development are shown in **Figure** **S1**. In the absence of inhibitors, GCDCA‐S plasma baseline concentration is approximately 0.08 μM (80 nM). In the studies conducted by Tatosian *et al*.^18^ (study #1) and Willemin *et al*.^22^ (study #2 and #3), the plasma baseline levels of GCDCA‐S exhibited moderate to high inter‐individual (IIV) (CV: 34%, 34%, and 69% respectively), as well as intra‐individual (diurnal variation) (CV: 34%, 35%, and 43%, respectively) variability. These values were greater than those reported for other biomarkers, namely CP‐I (IIV: CV < 25%)^6^ and PDA (IIV: CV = 27%).^7^

### Model development

**Figure 3:**
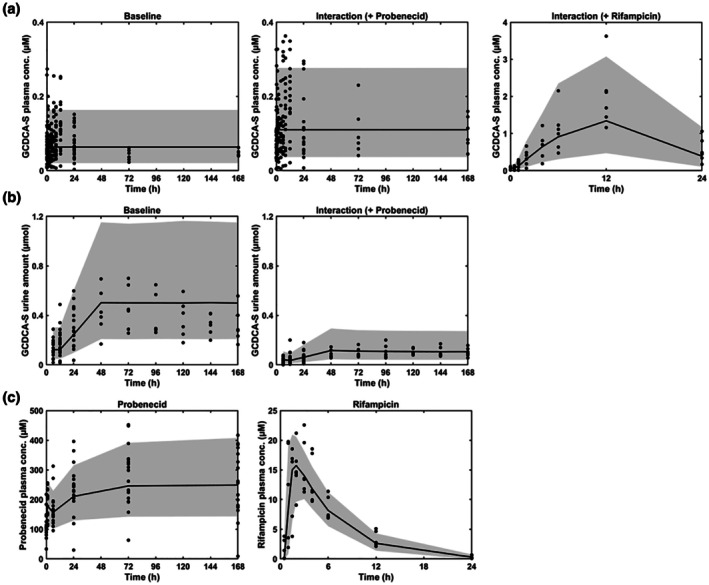
Visual predictive checks of the developed population PK models for GCDCA‐S, probenecid, and rifampicin. (**a**) GCDCA‐S plasma data in the absence and presence of inhibitors (probenecid or rifampicin), (**b**) GCDCA‐S urine data in the absence and presence of probenecid, (**c**) probenecid or rifampicin plasma data. Shaded area represents the 90% prediction interval (based on 5,000 simulated individuals in each group), black circles (●) are the observed literature data as listed in **Table** **S1**. Conc, concentration.

The structural models used to describe plasma concentrations of rifampicin and probenecid, and plasma/urine data for GCDCA‐S are shown in **Figure** **2**. Model parameters were successfully estimated for rifampicin, probenecid, and GCDCA‐S (**Table** **2**). The final model’s VPC and GOF plots are shown in **Figure** **3** and **Figure** **S2**, respectively.

**Table 2:** Parameter estimates of the population PK models for probenecid, rifampicin and GCDCA‐S.

Drugs		Parameter (units)	Estimates (RSE%)	
			Population^a^	IIV^b^
PROB	System parameters	*ka*_*PROB*_ (/h)	1.1 (37)	–
		*V*_*PROB*_ (L)	17 (10)	31 (28)
		*CL*_*PROB*_ (L/h)	0.95 (7)	27 (20)
		*K*_*i,unbound,OAT3*_ (μM)	2.7 (12)	32 (27)
		*X*	1.7 (5)	–
	Residual unexplained variabilities	*σ*_*prop*_ – *PROB* (%)	23 (8)	–
		*σ*_*add*_ – *PROB* (μM)	0.001 (FIXED)	–
RIF	System parameters	*Tlag*_*RIF*_ (h)	0.56 (20)	46 (32)
		*Tk0*_*RIF*_ (h)	0.84 (41)	77 (37)
		*V*_*RIF*_ (L)	35 (5)	–
		*CL*_*RIF*_ (L)	6.7 (8)	18 (35)
		*K*_*i,unbound,OATP1B3*_ (μM)	0.009 (29)	–
	Residual unexplained variabilities	*σ*_*prop*_ – *RIF* (%)	20 (14)	–
		*σ*_*add*_ – *RIF* (μM)	0.2 (FIXED)	–
GCDCA‐S	System parameters	*k*_*syn*_ (μmol/h)	1.0 (18)	61 (15)
		*V*_*c,GCDCA–S*_ (L)	4.8 (FIXED)	–
		*CL*_*R,GCDCA–S*_ (L/h)	0.31 (8)	24 (25)
		*CL*_*h,GCDCA–S*_ (L/h)	15 (13)	–
	Residual unexplained variabilities	*σ*_*prop*_ – GCDCA‐S plasma (%)	45 (3)	–
		*σ*_*prop*_ – GCDCA‐S urine (%)	29 (1)	–

GCDCA‐S, glycochenodeoxycholic acid 3‐O‐sulfate; IIV, Inter‐individual variability; PROB, probenecid; RIF, rifampicin.

^a^ The population (fixed effect) parameters and relative standard errors (RSE, %).

^b^ Estimated IIV (%) and its RSE (%).

#### Rifampicin and probenecid population PK models

The POP‐PK parameters for rifampicin and probenecid models are listed in **Table** **2**. For both drugs, the additive components of the combined error models were fixed. In the rifampicin model, inter‐individual variability (IIV) was estimated for *T*_lag_, *T*_K0_, and CL_rif_, with values of 46%, 77%, and 18%, respectively. The estimated IIV for *V*_prob_ and CL_prob_ was 31% and 27%, respectively. The rifampicin and probenecid model parameters were consistent with the values reported in other studies, where the same plasma clinical DDI data were utilized for POP‐PK analysis.^7^, ^33^ Both models adequately described the observed data, capturing both the median and the variability in the data (**Figure** **3**).

#### GCDCA‐S population PK model

The POP‐PK parameters for the GCDCA‐S model were successfully estimated, with reasonable standard errors (<40%) (**Table** **2**). The results indicated biliary excretion as the primary route of elimination for GCDCA‐S (>95%). The residual error for both plasma and urine GCDCA‐S data were described by a proportional residual error model. The estimated IIV for *k*_syn_ and CL_R_ was 61% and 24%, respectively. For model identifiability, *V*_*c*_ was fixed at 4.8 L, the value initially estimated from the POP‐PK model. The model successfully described the observed GCDCA‐S plasma and urine data, including both baseline conditions and the interaction phase (**Figure** **3**). To assess the diurnal variation of the GCDCA‐S plasma baseline, six different functions were investigated and incorporated into *k*_syn_. However, these functions did not improve model performance (*P* > 0.9) due to (i) lack of consistent patterns across subjects and (ii) unknown timing of food intake (**Table** **S2**). In addition, a sex covariate was added to *k*_syn_ of GCDCA‐S to evaluate its impact on GCDCA‐S plasma levels. Due to the limited sample size and high baseline variability, the model’s performance showed no improvement (*P* > 0.9), so this covariate was excluded from the final model (**Table** **S3**).

### GCDCA‐S model verification

**Figure 4:**
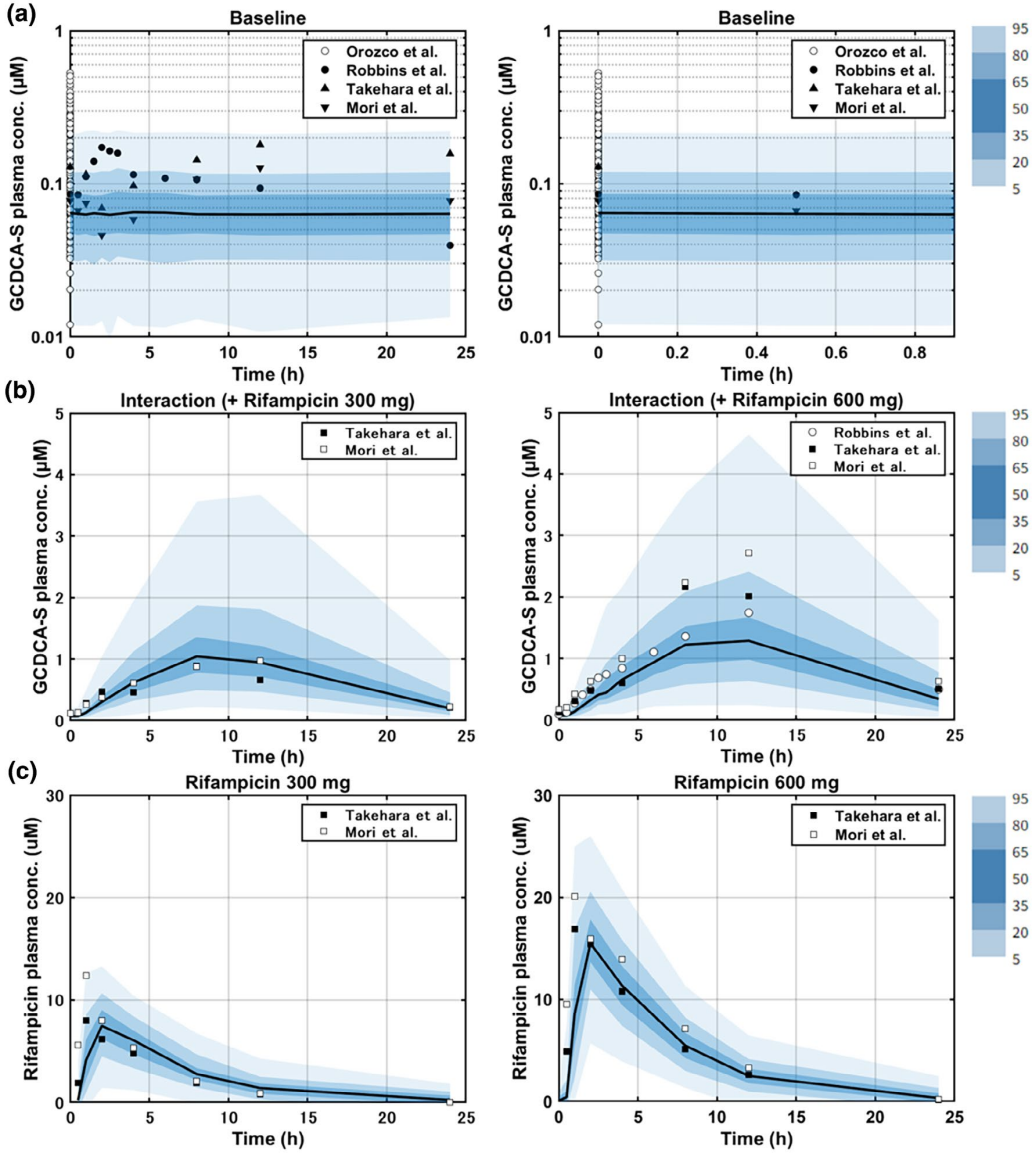
Verification of GCDCA‐S and rifampicin models against independent clinical data. (**a**) GCDCA‐S plasma baseline data (full time profile and 0–1 h time range). Baseline data in Orozco et al.^36^ was assigned as time 0 h points. (**b**) GCDCA‐S plasma data in the presence of rifampicin (300 and 600 mg) (**c**) rifampicin plasma data (300 and 600 mg). Shaded area represents 90% prediction interval (based on 5,000 simulated individuals in each group), circles, triangles, and squares are the observed literature data. Conc, concentration.

The predictive performance was evaluated using GCDCA‐S plasma baseline and interaction profiles following rifampicin administration from four independent studies (**Table** **S1**). Observations from a large‐scale clinical trial (Study #4), which included subjects with the *SLCO1B1* c.521T>C genetic polymorphism, were predicted within the 90% confidence interval. The majority of observed plasma baseline concentration‐time profiles from one clinical study in White (Study #5) and two clinical studies in Japanese (Study #6 and #7) were also reproduced within the 90% confidence interval of the simulated profiles. Furthermore, the model successfully captured the observed GCDCA‐S plasma interaction profiles following the administration of 300 mg and 600 mg rifampicin in White (Study #5) and Japanese (Study #6 and #7), as evidenced by the VPC plots where the majority of the observations were within the 90% prediction intervals (**Figure** **4**). However, while the model accurately predicted the GCDCA‐S AUCR following the administration of 600 mg rifampicin (predicted AUCR of 13 vs. the observed AUCR: 10–16),^15^, ^16^, ^17^ it tended to overestimate the AUCR after 300 mg rifampicin administration (predicted AUCR: 9.3, observed AUCR: 4.3–5.9).^15^, ^16^

### Power calculation

**Figure 5:**
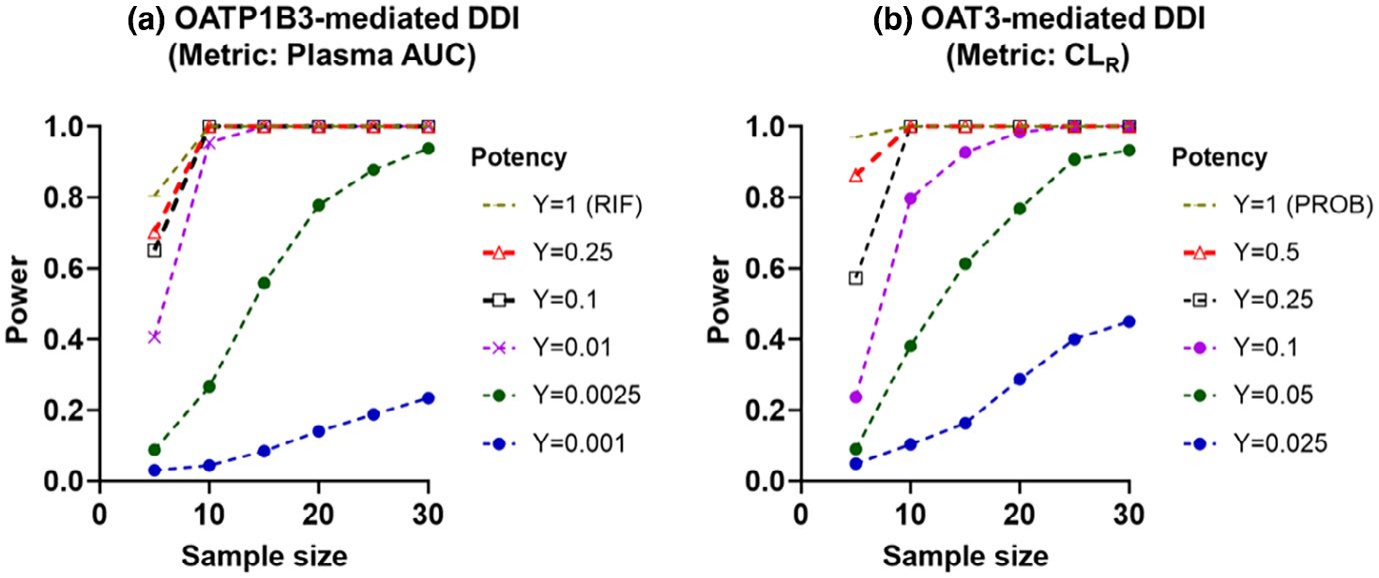
Power curves at significance levels (*α* = 0.01) for the different hypothetical I/Ki ratios (Y) based on a one‐sample paired t‐test of the ratio of logarithmic transformed AUC or CL_R_. (**a**) Power to detect OATP1B3‐mediated DDIs based on changes in GCDCA‐S plasma AUC, with I/Ki ratios ranging from 0.001 to 1 relative to that of rifampicin. (**b**) Power to detect OAT3‐mediated DDIs based on changes in GCDCA‐S CL_R_, with I/Ki ratios ranging from 0.01 to 1 relative to that of probenecid. Ratios of 1 correspond to equivalent I/Ki ratio of rifampicin or probenecid, respectively.

The predictive performance of the population PK model confirmed the utility of GCDCA‐S as an endogenous biomarker for identifying strong OATP1B3 and OAT3 inhibitors. Currently, there is a lack of clinical data on the effects of moderate and weak inhibitors on GCDCA‐S plasma/urine levels. To address this gap, GCDCA‐S plasma and urine profiles were simulated across different exposure levels and inhibitor potencies (I/Ki) ratios relative to that of rifampicin and probenecid, ranging from 0.001 to 1 for OATP1B3 and 0.01 to 1 for OAT3 inhibition. Simulated plasma AUC of GCDCA‐S following OATP1B3 inhibition was highly sensitive to the OATP1B3 I/K_i_ ratios, whereas the effect on its CL_R_ was negligible. In contrast, CL_R_ of GCDCA‐S was highly sensitive to the OAT3 inhibition, with minimal impact on its plasma AUC (**Figure** **S3**). Power calculations identified that 5 subjects are sufficient in the clinical trial to detect a strong OATP1B3‐mediated DDI (I/Ki ratio = 1 relative to rifampicin) by monitoring the increase in GCDCA‐S plasma AUC. In the case of moderate interaction (predicted AUCR >2, I/Ki = 0.25 relative to rifampicin) and weak interaction (1.25 < AUCR <2, I/Ki ratio = 0.05 relative to rifampicin) 10 subjects were needed. For detecting OAT3‐mediated DDI by monitoring GCDCA‐S CL_R_, 5, 10, and 15 subjects were required for strong, moderate, and weak inhibitors, respectively (**Figure** **5** and **Table** **S4**). Power calculations at a significance level of 0.05 are shown in **Figure** **S4**.

### Potential effect of transporter inhibitor on synthesis rate

Hypothetical simulations were conducted to evaluate the potential impact of rifampicin and probenecid on GCDCA‐S *k*_syn_. Plasma AUC in the presence of an inhibitor of synthesis was highly sensitive to changes in *k*_syn_, irrespective of the transporter affected. However, the impact on CL_R_ remained minimal (**Figure** **S5**). These simulations highlight the potential bias in the interpretation of biomarker‐informed DDI risk assessment if biomarker synthesis is also affected by transporter inhibitors.

## DISCUSSION

Endogenous biomarkers are promising tools for evaluating *in vivo* transporter function and assessing transporter‐mediated DDIs early in drug development. GCDCA‐S has been identified as a sensitive endogenous biomarker of hepatic OATP1B3 and renal OAT3 transporters.^1^ However, currently there are no mechanistic models for this biomarker. Therefore, the primary aim of this study was to quantify the synthesis rate of GCDCA‐S, as well as to delineate the relative contribution of the renal and hepatobiliary pathways to systemic elimination of GCDCA‐S.

### GCDCA‐S model development and verification

GCDCA‐S is a bile acid sulfate conjugate derived from GCDCA, the glycine‐conjugated form of CDCA, a primary bile acid biosynthesized in the liver from cholesterol. The rate of synthesis of CDCA, which is mediated by CYP7A1, is widely considered to be the rate‐limiting step to the synthesis of all related bile acids.^24^, ^43^, ^44^ The rate of SULT2A1‐mediated sulfation of GCDCA has not been studied; therefore, the synthesis rate of GCDCA‐S was assumed to be constrained by the synthesis rate of its precursor bile acids (CDCA and GCDCA). The model estimated *k*_syn_ for GCDCA‐S was in agreement with reported *k*_syn_ values for its precursor^43^, ^44^, ^45^, ^46^ (**Table** **S5**). This study is the first to successfully quantify the synthesis rate of GCDCA‐S.

The current modeling analysis indicated biliary excretion as the primary route of elimination for GCDCA‐S (~95%). The estimated fraction excreted in urine (*f*_*e*_) of GCDCA‐S (<5%) was consistent with literature reports that both unsulfated and sulfated bile acids are thought to be excreted predominantly via biliary excretion.^29^, ^30^, ^32^, ^47^ For example, radio‐labeled studies in healthy human volunteers reported that *f*_*e*_ of lithocholic acid sulfate was <5%.^29^, ^30^, ^32^, ^47^ In a pre‐clinical study that characterized the PK of GCDCA‐S in bile duct‐cannulated monkeys, the reported *f*_*e*_ of GCDCA‐S was <1%.^19^ The estimated values of *f*_*e*_ and CL_h_ from the current modeling work were consistent with those inferred from the mean profiles of other reported clinical DDI studies (**Table** **S6**). The finding of low *f*_*e*_ aligns with clinical reports in chronic kidney disease patients where no significant difference in GCDCA‐S plasma concentrations (baseline and interaction profile after rifampicin administration) was noted relative to healthy individuals.^18^

The POP‐PK modeling also allowed the quantification of the *in vivo* inhibition potency of rifampicin and probenecid against OATP1B3 and OAT3, respectively, using GCDCA‐S as a substrate. The estimated *in vivo K*_*i*,u,OATP1B_ of 0.009 μM was slightly lower than the *in vivo K*_*i*,u,OATP1B_ values (0.020–0.14 μM) reported with CP‐I as a substrate (**Table** **S7**).^6^, ^8^, ^9^, ^11^, ^13^, ^33^, ^48^ It is important to note that the primary transporter involved in the hepatic uptake of GCDCA‐S is OATP1B3, whereas CP‐I relies on OATP1B1^1^, ^2^, ^28^; therefore, these *in vivo K*_*i*_ values correspond to the dominant transporter for each probe. These findings align with *in vitro* data showing that rifampicin *K*_*i*,OATP1B3_, based on GCDCA‐S as a substrate, was lower than the *K*_*i*,OATP1B1_ with CP‐I as a substrate^28^ (**Table** **S7**). Furthermore, a potential increase in *k*_syn_ due to rifampicin administration may confound the estimate of *K*_*i*,u,OATP1B3_. However, currently available data were insufficient to incorporate the effect of rifampicin on GCDCA‐S synthesis into the model. Hypothetical simulations indicated that changes in *k*_syn_ would strongly affect GCDCA‐S plasma exposure (**Figure** **S5**), and therefore, the observed AUCR and associated DDI risk classification may be biased. The estimated *in vivo K*_*i*,u,OAT3_ was in agreement with previously reported *in vivo* inhibition potencies of probenecid against OAT1/3 based on PDA data.^7^ GCDCA‐S is a sensitive endogenous biomarker for the risk assessment of OAT3‐mediated DDIs, based on fold reduction in renal clearance as a metric for DDI classification.

The current GCDCA‐S model did not consider enterohepatic recirculation of GCDCA‐S, as bile acid sulfates undergo limited enterohepatic recirculation due to their limited absorption in the intestine.^29^, ^30^, ^31^ Furthermore, pre‐clinical studies reported that the enterohepatic recirculation had minimal effect on GCDCA‐S plasma concentrations in the short period (<8 h).^19^ Over a long time range, enterohepatic circulation of precursor (GCDCA) may influence the systemic profile of GCDCA‐S. Due to the lack of precursor (GCDCA) data, any potential influence of enterohepatic recirculation was not incorporated into the POP‐PK model.

### Design of clinical studies monitoring GCDCA‐S

Power calculations have confirmed the adequacy of the design used in previous clinical DDI studies with rifampicin (6–8 subjects^14^, ^15^, ^16^, ^17^, ^18^) and probenecid (6–8 subjects^22^, ^23^), and the suitability of GCDCA‐S as an endogenous biomarker for the evaluation of strong OATP1B3 and OAT3 inhibitors. Power calculations for different hypothetical OATP1B3 and OAT3 inhibitors were performed assuming that these inhibitors follow the same PK as rifampicin or probenecid, respectively. In the case of OATP1B3‐mediated DDIs, 5–10 subjects were required to detect strong and moderate inhibitors, and this sample size was comparable to CP‐I as a biomarker.^6^ In contrast, GCDCA‐S showed greater sensitivity in detecting weak OATP1B3 inhibition, needing only 10 individuals, compared with CP‐I data that requires 15 subjects for the same magnitude of interaction^6^ (**Figure** **5** and **Table** **S4**). Although GCDCA‐S exhibits higher baseline variability compared to CP‐I, the changes in GCDCA‐S plasma AUC after the administration of OATP1B3 inhibitors were more pronounced (AUCR_0–24h_ = 10.8 (GCDCA‐S) vs. 4.3 (CP‐I) after rifampicin 600 mg administration),^18^ supporting its ability to detect weak DDIs even with a smaller sample size. These findings highlight the utility of monitoring GCDCA‐S plasma AUC as a Tier 2 biomarker alongside CP‐I plasma AUC to delineate between OATP1B1 and OATP1B3‐mediated DDIs. The required sample size for detecting OAT3‐mediated DDIs (5–15 subjects for strong to weak inhibitors) is similar to OATP1B3, although CL_R_ is used as the metric in this case instead of plasma AUC. These requirements were comparable to PDA,^7^ highlighting that OAT3‐mediated DDIs can be detected by monitoring not only PDA plasma AUC,^10^, ^22^ but also GCDCA‐S CL_R_ (by monitoring plasma GCDCA‐S and amounts excreted in urine).^22^ A potential framework for leveraging GCDCA‐S interaction data in plasma and urine is presented in **Figure** **S6**. At this stage, the framework serves as a qualitative guidance and lacks definitive cut‐off values to support the decision‐making process. To establish such values, GCDCA‐S data with OATP1B3/OAT3 inhibitors with varying potencies are necessary, as has been accomplished for CP‐I.^1^, ^3^ The frameworks also highlight the necessity to investigate whether the drug alters the *k*_syn_ of GCDCA‐S, to avoid bias in the interpretation of plasma AUCR data. Unlike CP‐I, reliance on a single point (*C*_max_R) or a short time‐span AUCR (e.g., AUCR_0–4h_) may lead to misinterpretation of DDI risk using GCDCA‐S data. Monitoring middle‐term AUCR (e.g., AUCR_0–8h_, AUCR_0–12h_) under fasting conditions would be ideal; however, this may not be practical. Therefore, AUC_0–24h_ under fed conditions may be the most feasible option, as it can better reflect overnight plasma levels when the baseline tends to be more stable.

In conclusion, this study developed a GCDCA‐S population PK model to support further its validation as an endogenous biomarker of OATP1B3 and OAT3 transporters. The power calculation conducted in this study provides a valuable resource for optimizing the design of prospective OATP1B3 and OAT3 DDI studies in early‐phase clinical trials. GCDCA‐S has shown greater sensitivity for detecting OATP1B3‐mediated DDIs, requiring a smaller sample size than CP‐I, whereas for OAT3‐mediated DDIs, the required sample size was comparable to PDA. The successful quantification of the synthesis rate and systemic elimination pathways of GCDCA‐S is critical for the development of a PBPK model for this biomarker, and future robust evaluation of OATP1B3‐ and OAT3‐mediated DDIs using GCDCA‐S data.

## FUNDING

Y.U. was financially supported by a fellowship grant from Asahi Kasei Pharma Corporation.

## CONFLICT OF INTEREST

The authors declared no competing interests for this work.

## AUTHOR CONTRIBUTIONS

Y.U., V.G., K.O., and A.G. wrote the manuscript. Y.U., V.G., K.O., and A.G. designed the research. Y.U., and V.G. performed the research. Y.U., and V.G. analyzed the data.

## Supporting information


Data S1.
