# Supplementary material for: Population Pharmacokinetic Modeling of Glycochenodeoxycholic Acid 3‐O‐Sulfate (GCDCA‐S) as Endogenous Biomarker of OATP1B3 and OAT3 Transporters
Source: Clin Pharmacol Ther. 2025 Aug 11;118(6):1532–42. doi: 10.1002/cpt.70023 (PMC12641067; doi:10.1002/cpt.70023)
Supplement: Supplementary file 1 — Data S1. [file CPT-118-1532-s001.pdf]

Supplementary Material

Population pharmacokinetic modelling of glycochenodeoxycholic acid 3-O-sulfate (GCDCA-S) as endogenous biomarker of OATP1B3 and OAT3 transporters

Yuki Ujihira[1,2], Viktor Georgiev[1], Kayode Ogungbenro[1] and Aleksandra Galetin[1]

[1] Centre for Applied Pharmacokinetic Research, School of Health Sciences, University of Manchester, Manchester, UK

[2] Laboratory for Safety Assessment and ADME, Pharmaceuticals Research Center, Asahi Kasei Pharma Corporation, Shizuoka, Japan

Table S1. Clinical data used for GCDCA-S population PK model verification

	Study #4 (Orozco *et al.*[1])	Study #5 (Robbins *et al.*[2]) [b]	Study #6 (Takehara *et al.*[3])	Study #7 (Mori *et al.*[4])
Ethnicity	Finnish White	White, Black or African American	Japanese	Japanese
Sex (No. subjects)	Male (173), Female (183)	Female (6)	Male (8)	Male (8)
Age range (year)	24.1 (mean)	23-52	26-36	26-36
Duration of sample collection (h)	1 point	24	24	24
Food intake	Fasting plasma sample	participants fasted from ≥ 10 hours predose until ≥ 4 hours postdose	4h and 10h post dose	4h and 10h post dose
Inhibitor	No inhibitor	RIF	RIF	RIF
No. occasions; treatment	pretreatment	OCC1; Letermovir<br>OCC2; Letermovir + RIF 600mg	OCC1; Placebo<br>OCC2; Statin<br>OCC3; Statin + RIF 300mg<br>OCC4; Statin + RIF 600mg	OCC1; Placebo<br>OCC2; Probe cocktail<br>OCC3; Probe cocktail + RIF 300mg<br>OCC4; Probe cocktail + RIF 600mg
$C_{baseline}$ (nM)	122 [a]	115 [c]	150 [c]	90 [c]

$C_{baseline}$, GCDCA-S plasma baseline concentration in the absence of inhibitors. [a] Plasma concentration of subjects with *SLCO1B1* c.521TT genotype were used for model verification. [b] In both occasions, Letermovir 480mg was administered, and it has been reported that single-dose Letermovir led to small increases in GCDCA-S concentrations vs. predose levels. [c] Calculated based on the reported plasma AUC_{0-24h}.

Evaluation of diurnal fluctuation in GCDCA-S plasma concentrations

To represent diurnal fluctuations, we evaluated six different functions: Bateman, Inverse Bateman, Power, Polynomial, Exponential, and Fourier functions. These are commonly used methods in population PK modelling to capture variability and can also describe multiple peaks observed in GCDCA-S concentrations that are influenced by food intake. However, in the clinical data used in this study, these functions did not improve model performance due to (1) lack of consistent patterns across subjects and (2) unknown timing of food intake.

$$\frac{dC_{GCDCA-S}}{dt} = \left[k_{syn}\,(1 + Function) - \left(C_{GCDCA-S} \times \frac{CL_h}{1 + \frac{C_{u,RIF}}{K_{i,u,OATP1B3}}} \times \frac{1}{X} + C_{GCDCA-S} \times \frac{CL_R}{1 + \frac{C_{u,PROB}}{K_{i,u,OAT3}}}\right)\right] \times \frac{1}{Vc} \qquad \textbf{(Eq.1)}$$

Table S2. Impact of diurnal fluctuation functions on model Fit (OFV)

Functions	Model structure	Parameter	OFV
Not defined	-	-	-642
Bateman	$P_{max}(e^{-k_{on}t} - e^{-k_{off}t})$	*P_{max}- amplitude*<br>*k_{on} – rate constant*<br>*k_{off} – rate constant*	-632
Inverse Bateman	$P_{max}(1 - (e^{-k_{on}t} - e^{-k_{off}t}))$	*P_{max}- amplitude*<br>*k_{on} – rate constant*<br>*k_{off} – rate constant*	-551
Power	$m \times t^p$	m – slope<br>p – power	-558
Polynomial	$m \times t + q \times t^2$	*m – slope*<br>*q – quadratic coefficient*	-548
Exponential	$P_{max}(1 - e^{-kt})$	*P_{max}- amplitude*<br>*k – rate constant*	-572
Fourier	$AMP_1 \cdot \cos(2\pi(t - \emptyset_1)/12) + AMP_2 \cdot \cos(2\pi(t - \emptyset_2)/6)$	*AMP1, AMP2 – amplitudes*<br>*Ø1, Ø2 - acrophase*	N.C.

OFV, -2 × log-likelihood; N.C., Not Converged

The effect of sex on GCDCA-S synthesis rate

In line with previous reports,[1,5–7] baseline plasma concentrations of GCDCA-S were higher in male subjects than in female among the 24 individuals included in the POPPK model development (Figure S1B). To account for this observation, the potential effect of sex on the GCDCA-S synthesis rate ($k_{syn,sex}$) was incorporated into the model using Equation 2, with sex implemented as a categorical covariate. However, the covariate was excluded from the final model, as the limited sample size and high baseline variability resulted in no improvement in model performance ($p > 0.9$; Table S3).

$$\boldsymbol{k_{syn,sex}} = \boldsymbol{k_{syn,male}} \times (\boldsymbol{1} - \boldsymbol{SEX} \times \boldsymbol{COV_{SEX}}) \qquad \textbf{(Eq.2)}$$

where COV_{SEX} is the fractional change in k_{syn} *in* women relative to men, and *SEX* is a dummy variable that takes the value of 0 for men and 1 for women.

Table S3. Parameter estimates of the population PK models for GCDCA-S

**Drugs**		**Parameter (units)**	**Without sex effect**		**With sex effect**	
			**Estimates (RSE%)**			
			**Population[a]**	**IIV[b]**	**Population[a]**	**IIV[b]**
GCDCA-S	System parameters	k_{syn} (μmol/h)	1.0 (18)	61 (15)	1.0 (18)	61 (15)
		$V_{c,\ GCDCAS}$ (L)	4.8 (FIXED)	-	4.8 (FIXED)	-
		$CL_{R,\ GCDCAS}$ (L/h)	0.31 (8)	24 (25)	0.31 (8)	24 (25)
		$CL_{h,\ GCDCAS}$ (L/h)	15 (13)	-	14 (13)	-
	Covariates	COV_{SEX}	-	-	0.065 (404)	-
OFV			-642		-639	

COV_{SEX}, fractional change in k_{syn} in women relative to men; OFV, -2 × log-likelihood
[a] The population (fixed effect) parameters and relative standard errors (RSE, %).
[b] Estimated IIV (%) and its RSE (%).

Power calculation

To evaluate the utility of GCDCA-S in identifying weak and moderate OATP1B3 and OAT3 inhibitors, GCDCA-S plasma and urine data were simulated under varying exposure levels and inhibitor potencies (I/Ki) ratios relative to rifampicin and probenecid, ranging from 0.001–1 for OATP1B3 (equations 3 and 4) and 0.01-1 for OAT3 inhibition (equations 5 and 6). Ratios of 1 indicate equivalence to rifampicin or probenecid. The classification of inhibitors may vary depending on the sensitivity of endogenous biomarker used. To evaluate the relative sensitivity of GCDCA-S compared to other biomarkers, OATP1B inhibitors were classified based on our previous report using changes in plasma AUC of CP-I,[8] whereas OAT3 inhibitors were classified according to changes in plasma AUC of PDA[9] (moderate inhibitors, predicted AUCR > 2; weak inhibitors, 1.25 < AUCR < 2) (Table S4).

(A) OATP1B3 inhibitors

$$\frac{dC_{GCDCAS}}{dt} = \left[k_{syn} - \left(C_{GCDCAS} \times \frac{CL_h}{1 + (\frac{C_{u,RIF}}{K_{i,u,OATP1B3}})'} + C_{GCDCAS} \times CL_R\right)\right] \times \frac{1}{Vc} \qquad \textbf{(Eq.3)}$$

$$\frac{dA_{GCDCAS}}{dt} = C_{GCDCAS} \times CL_R \qquad \textbf{(Eq.4)}$$

(B) OAT3 inhibitors

$$\frac{dC_{GCDCAS}}{dt} = \left[k_{syn} - \left(C_{GCDCAS} \times CL_h \times \frac{1}{X} + C_{GCDCAS} \times \frac{CL_R}{1 + (\frac{C_{u,PROB}}{K_{i,u,OAT3}})'}\right)\right] \times \frac{1}{Vc} \qquad \textbf{(Eq.5)}$$

$$\frac{dA_{GCDCAS}}{dt} = C_{GCDCAS} \times \frac{CL_R}{1 + (\frac{C_{u,\ PROB}}{Ki\ u\ OAT3})'} \qquad \textbf{(Eq.6)}$$

where $C_{GCDCA\text{-}S}$ (µM) is GCDCA-S plasma concentration, $A_{GCDCA\text{-}S}$ (µmol) is GCDCA-S amount in urine, k_{syn} (µmol/h) is the zero-order GCDCA-S synthesis rate, CL_h (L/h) is hepatic clearance of GCDCA-S, CL_R (L/h) is renal clearance of GCDCA-S, V_C (L) is the volume of distribution of GCDCA-S, $K_{i,u,OATP1B3}$ (µM) is unbound OATP1B3 inhibition constant for rifampicin, $C_{u,RIF}$ (µM) is unbound plasma concentration for rifampicin (linked to RIF model), $K_{i,u,OAT3}$ (µM) is unbound OAT3 inhibition constant for probenecid, $C_{u,PROB}$ (µM) is unbound plasma concentration for probenecid (linked to PROB model), and X is a fold reduction in hepatic clearance of GCDCA-S in the presence of probenecid.

Table S4. Classification of the inhibitory potency of the inhibitors used for the power calculation at significance levels (α=0.01).

I/Ki ratio relative to rifampicin	Classification of inhibitors in CP-I model[8]	Inhibitor dosing	CP-I		GCDCA-S	
			$AUCR_{0\text{-}24h}$	Required size	$AUCR_{0\text{-}24h}$	Required size
1	rifampicin (strong inhibitor)	600mg, SD	3.5	5	13	5
0.25	moderate inhibitor		> 2	10	6.9	10
0.05	weak inhibitor		> 1.25	15	3.1	10
**I/Ki ratio relative to probenecid**	**Classification of inhibitors in PDA model[9]**	**Inhibitor dosing**	**PDA**		**GCDCA-S**	
			$AUCR_{0\text{-}24h}$	Required size	CL_R ratio [a]	Required size
1	probenecid (strong inhibitor)	500mg, QID	3.9	5	0.1	5
0.25	moderate inhibitor		> 2	10	0.3	10
0.1	weak inhibitor		> 1.25	15	0.5	15

[a] $CL_{R,+inhibitor}$ / $CL_{R,control}$; CP-I, coproporphyrin-I; PDA, pyridoxic acid; SD, single dose; QID, quater in die.

Potential effect of transporter inhibitor on GCDCA-S synthesis rate

The potential effect of rifampicin and probenecid on the synthesis rate of GCDCA-S (in addition to their inhibition effects on OATP1B3 and OAT3 transporters) was investigated using the developed GCDCA-S model. Inhibitory effect of inhibitors on GCDCA-S k_{syn} was assessed using equation 7 and 8, which was previously applied to CP-I[8] and PDA[9] models. Potential increase effect of inhibitors on GCDCA-S k_{syn} was evaluated using equation 9 and 10. GCDCA-S plasma AUC and CL_R were simulated across different I/Ki ratios (0.01, 0.1 and 1) relative to inhibitory effect of rifampicin and probenecid on transporters.

(A) Assuming rifampicin decreases GCDCA-S k_{syn}

$$\frac{dC_{GCDCAS}}{dt} = \left[\frac{k_{syn}}{1+(\frac{C_{u,RIF}}{K_{i,u,OATP1B3}})'} - \left(C_{GCDCAS} \times \frac{CL_h}{1+\frac{C_{u,RIF}}{K_{i,u,OATP1B3}}} + C_{GCDCAS} \times CL_R\right)\right] \times \frac{1}{Vc} \quad \textbf{(Eq.7)}$$

(B) Assuming probenecid decreases GCDCA-S k_{syn}

$$\frac{dC_{GCDCAS}}{dt} = \left[\frac{k_{syn}}{1+(\frac{C_{u,PROB}}{K_{i,u,OAT3}})'} - \left(C_{GCDCAS} \times CL_h \times \frac{1}{X} + C_{GCDCAS} \times \frac{CL_R}{1+\frac{C_{u,PROB}}{K_{i,u,OAT3}}}\right)\right] \times \frac{1}{Vc} \quad \textbf{(Eq.8)}$$

(C) Assuming rifampicin increases GCDCA-S k_{syn}

$$\frac{dC_{GCDCAS}}{dt} = \left[k_{syn} \times (1 + \left(\frac{C_{u,RIF}}{K_{i,u,OATP1B3}}\right)') - \left(C_{GCDCAS} \times \frac{CL_h}{1+\frac{C_{u,RIF}}{K_{i,u,OATP1B3}}} + C_{GCDCAS} \times CL_R\right)\right] \times \frac{1}{Vc} \quad \textbf{(Eq.9)}$$

(D) Assuming probenecid increases GCDCA-S k_{syn}

$$\frac{dC_{GCDCAS}}{dt} = \left[k_{syn} \times (1 + \left(\frac{C_{u,PROB}}{K_{i,u,OAT3}}\right)') - \left(C_{GCDCAS} \times CL_h \times \frac{1}{X} + C_{GCDCAS} \times \frac{CL_R}{1+\frac{C_{u,PROB}}{K_{i,u,OAT3}}}\right)\right] \times \frac{1}{Vc} \quad \textbf{(Eq.10)}$$

where $C_{GCDCA\text{-}S}$ (µM) is GCDCA-S plasma concentration, k_{syn} (µmol/h) is the zero-order GCDCA-S synthesis rate, CL_h (L/h) is hepatic clearance of GCDCA-S, CL_R (L/h) is renal clearance of GCDCA-S, V_C (L) is the volume of distribution of GCDCA-S, $K_{i,u,OATP1B3}$ (µM) is unbound OATP1B3 inhibition constant for rifampicin, $C_{u,RIF}$ (µM) is unbound plasma concentration for rifampicin (linked to RIF model), $K_{i,u,OAT3}$ (µM) is unbound OAT3 inhibition constant for probenecid, $C_{u,PROB}$ (µM) is unbound plasma concentration for probenecid (linked to PROB model), and X is a fold reduction in hepatic clearance of GCDCA-S in the presence of probenecid.

Verification of GCDCA-S k_{syn} estimated in this study

Literature data on the SULT2A1-mediated sulfation rate of GCDCA were not available. To evaluate the validity of GCDCA-S k_{syn} estimated by the POPPK model, GCDCA-S k_{syn} was assumed to be constrained by the synthesis rate of its precursor bile acids, and an inference was made based on the reported k_{syn} value of the corresponding precursors (CDCA and GCDCA).

In the absence of an inhibitor, k_{syn} of bile acids in the turnover model is represented by Equation 11. Under steady-state conditions (where *dC/dt=0*), and given that the values of CL_R of GCDCA-S and its corresponding precursors are relatively small compared to their CL_h, k_{syn} of bile acids is expressed by Equation 12.[10,11] It has been reported that the biliary excretion of bile acid sulfates is slower than that of their unsulfated forms,[10,12] and assuming $CL_{h,GCDCA\text{-}S} < CL_{h,precursors}$, GCDCA-S k_{syn} was inferred, as shown in Table S5.

$$\frac{dC_{bile\ acid}}{dt} = \left[k_{syn} - \left(C_{bile\ acid} \times CL_h + C_{bile\ acid} \times CL_R\right)\right] \times \frac{1}{Vc} \qquad \textbf{(Eq.11)}$$

$$k_{syn} = C_{bile\ acid} \times CL_h \qquad \textbf{(Eq.12)}$$

where *C* (µM) is plasma concentration, k_{syn} (µmol/h) is the zero-order synthesis rate, CL_h (L/h) is hepatic clearance, CL_R (L/h) is renal clearance, V_C (L) is the volume of distribution.

Table S5. GCDCA-S k_{syn} inferred from the k_{syn} of its corresponding precursors

Biomarker	Plasma baseline (nM)		Denovo k_{syn} (µmol/h)		
	Reported value	Ref	Reported value	Ref	Notes
CDCA	510 [a]	4	18 [b]	13,14	Information 1
			17 [c]	15	Information 2
GCDCA	540 [a]		14 [d]	15	Information 3
**Biomarker**	**Plasma baseline (nM)**		**Inferred k_{syn} (µmol/h)**		
GCDCA-S	85 [a]	4	< 3.0	Based on information 1	
			< 2.8	Based on information 2	
			< 2.2	Based on information 3	

[a]Averaged C_{mean} value of the baseline and control groups. [b]Synthesis rate of unconjugated chenodeoxycholic acid. [c]Averaged synthesis rate of chenodeoxycholic acid reported in six studies. [d]Synthesis rate of chenodeoxycholic acid-glycine.

Verification of GCDCA-S f_e estimated in this study

The validity of GCDCA-S CL_h and renal excretion fraction (f_e) estimated in this study was evaluated by incorporating the GCDCA-S mean plasma concentration profile in other reported clinical DDI studies into the developed POPPK models. In the absence of inhibitors, under steady-state conditions (where $dC/dt=0$), GCDCA-S k_{syn} is expressed as shown in Equation 13. In the presence of rifampicin, at $t=T_{max}$ (where $dC/dt=0$), GCDCA-S k_{syn} is expressed as shown in Equation 14. By solving equation 13 and 14, CL_h can be derived as shown in Equation 15. The CL_h and f_e values inferred from data obtained in each clinical DDI study are summarized in Table S6.

$$\boldsymbol{k_{syn} = C_{baseline} \times CL_h + C_{baseline} \times CL_R} \qquad \textbf{(Eq.13)}$$

$$\boldsymbol{k_{syn} = C_{max} \times \frac{CL_h}{1+\frac{C_{u,RIF}}{K_{i,u,OATP1B3}}} + C_{max} \times CL_R} \qquad \textbf{(Eq.14)}$$

$$\boldsymbol{CL_h = CL_R \times \frac{C_{max}-C_{baseline}}{C_{baseline} - C_{max} \times \frac{1}{(1+\frac{C_{u,RIF}}{K_{i,u,OATP1B3}})}} > CL_R \times \frac{C_{max}-C_{baseline}}{C_{baseline}}} \qquad \textbf{(Eq.15)}$$

where $C_{baseline}$ (µM) is plasma baseline concentration, C_{max} (µM) is maximum plasma concentration, k_{syn} (µmol/h) is the zero-order synthesis rate, CL_h (L/h) is hepatic clearance, CL_R (L/h) is renal clearance, $C_{u,RIF}$ (µM) is unbound plasma concentration of rifampicin, $K_{i,u,OATP1B3}$ (µM) is unbound inhibition constant against OATP1B3 for rifampicin.

Table S6. GCDCA-S CL_h and fe inferred from other reported clinical DDI studies

**Reported values**			**Inferred values**		**Ref**
**$C_{baseline}$[a] (µM)**	**C_{max} (µM)**	**CL_R[b] (L/h)**	**CL_h (L/h)**	**fe (%)**	
0.12	1.7	0.37	> 5.2	< 6.6%	[2]
0.12	3.8		> 12	< 3.1%	[16]
0.15	2.2		> 5.0	< 6.9%	[3]
0.09	2.7		> 11	< 3.3%	[4]

$C_{baseline}$, plasma baseline concentration of GCDCA-S; C_{max}, maximum plasma concentration of GCDCA-S; f_e, fraction excreted in urine of GCDCA-S; CL_h, hepatic clearance of GCDCA-S; CL_R, renal clearance of GCDCA-S.

[a] Average concentration from 0 to 24hours. [b] The CL_R value reported by Willemin *et al.*[17] was used for calculation. Even when using the reported CL_R values from other studies, the corresponding increase in CL_h ensures that the estimated f_e value in this calculation remains unchanged.

Table S7. Summary of *in vitro* and estimated/reported *in vivo* K_i values for rifampicin and probenecid

**Inhibitor**	**Substrate**	**Cell line**	***In vitro* OATP1B1 IC_{50} (μM)**	***In vitro* OATP1B3 IC_{50} (μM)**	**Reference**
RIF	GCDCA-S	HEK293	0.17	0.28	[18]
	CP-I	HEK293	0.61	0.57	[18]
**Inhibitor**	**Substrate**	**Inhibitor dose (mg)**		***In vivo* OATP1B $K_{i,u}$ (μM)**	**Reference**
RIF	GCDCA-S	600		0.009 (OATP1B3)	Current study
	CP-I	150 – 600		0.020 – 0.14 (OATP1B or 1B1)	Reported range
		600		0.13	[8]
		600		0.020	[19]
		300 - 600		0.082 - 0.11	[20]
		600		0.10	[21]
		150 – 600		0.14 (OATP1B1)	[22]
		600		0.038	[23]
		600		0.053	[24]
**Inhibitor**	**Substrate**	**Cell line**	***In vitro* OAT1 IC_{50} (μM)**	***In vitro* OAT3 IC_{50} (μM)**	
PROB	GCDCA-S	HEK293	N.R.	7.4	[25]
	PDA	HEK293	22	4.2	[9]
	HVA	N.R.	N.R.	N.R.	-
**Inhibitor**	**Substrate**	**Inhibitor dose (mg)**		***In vivo* OAT1/3 $K_{i,u}$ (μM)**	
PROB	GCDCA-S	500, QID		2.7 (OAT3)	Current study
	PDA			3.5 (OAT1/3)	[9]
	HVA			8.4 (OAT1/3)	[9]

CP-I, coproporphyrin-I; IC_{50}, half maximal inhibitory concentration; HVA, homovanillic acid; $K_{i,u}$, unbound inhibition constant; PDA, pyridoxic acid; QID, Quater in die. N.R., not reported.

Supplementary Figures

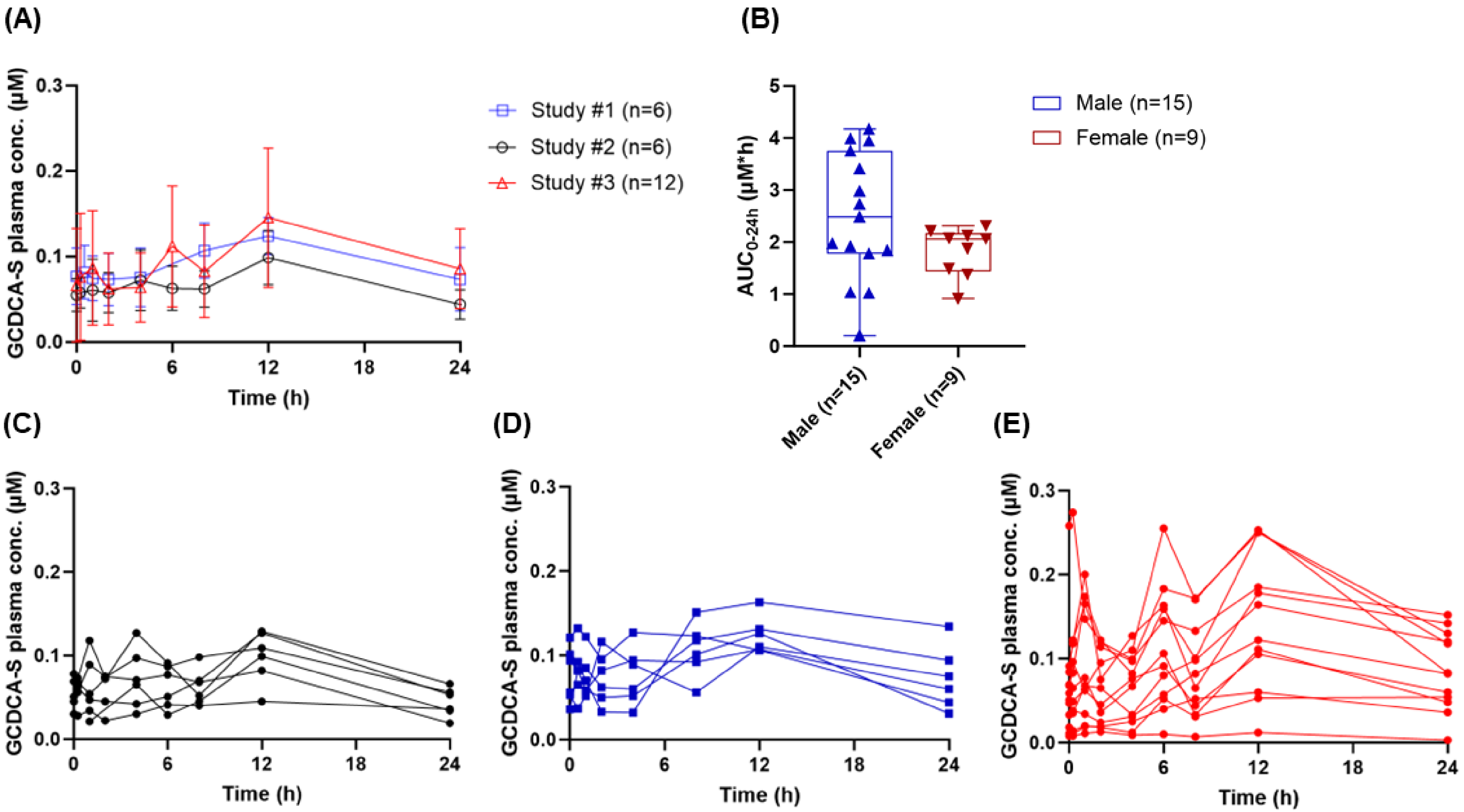

Figure S1. Variability in plasma concentrations of GCDCA-S in control phase. (A) Mean plasma baseline profiles across the three clinical studies (Study #1, #2 and #3). (B) Comparison of $AUC_{0\text{-}24h}$ values between White male and female subjects. (C-E) Individual plasma concentration profiles in Study #1 (C), #2 (D) and #3 (E), respectively.

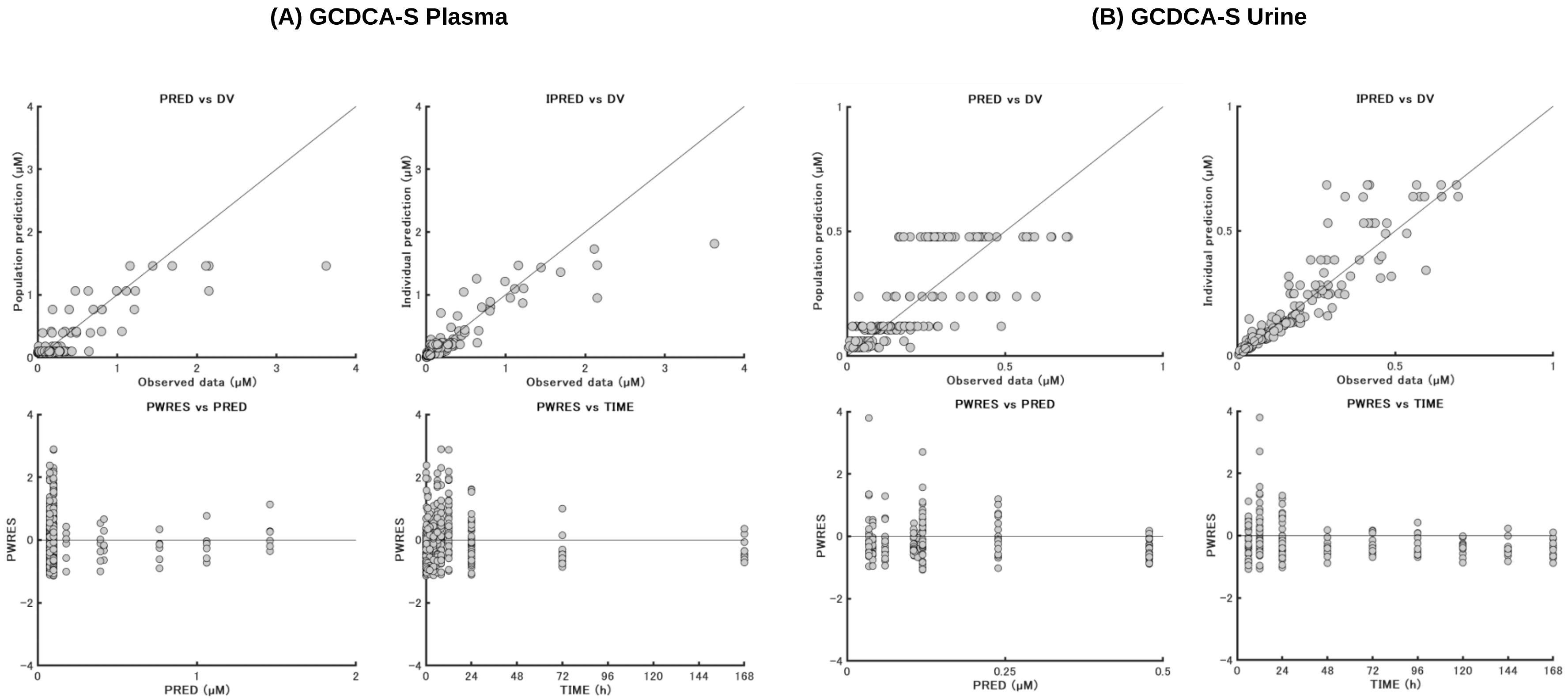

(A) GCDCA-S Plasma
(B) GCDCA-S Urine
PRED vs DV
IPRED vs DV
PWRES vs PRED
PWRES vs TIME
Population prediction (μM)
Individual prediction (μM)
Observed data (μM)
PWRES
PRED (μM)
TIME (h)

(C) RIF Plasma

(D) PROB Plasma

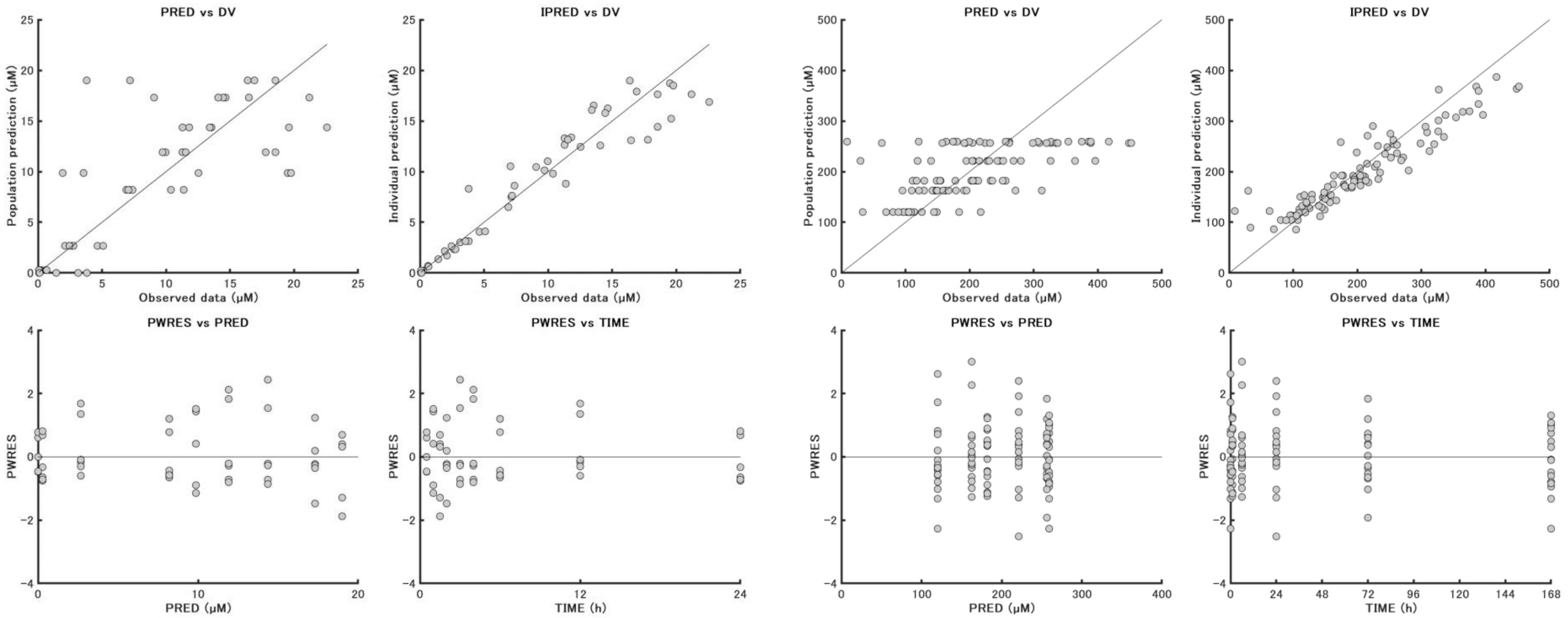

Figure S2. Goodness-of-fit (GOF) plots for population PK models. (A) GCDCA-S plasma data (B) GCDCA-S urine data (C) rifampicin plasma data (D) probenecid plasma data.

DV, observed data; PRED, population prediction; IPRED, individual prediction; PWRES, population weighted residuals. Solid lines are reference lines

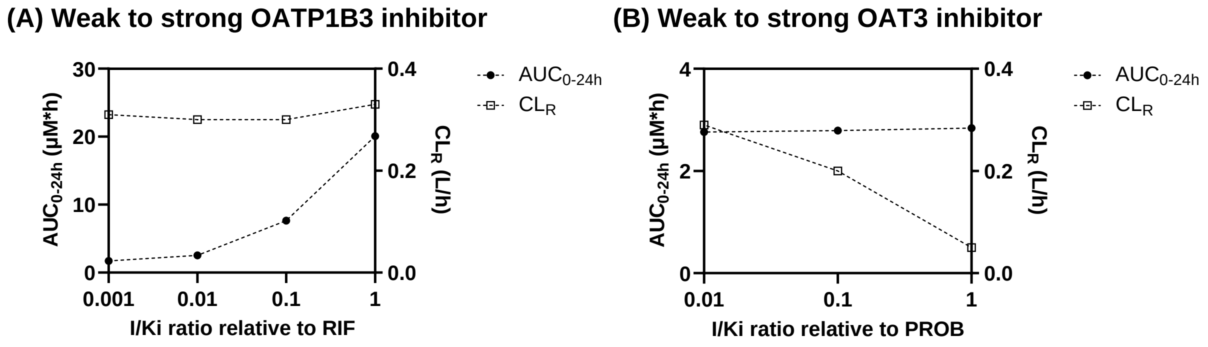

Figure S3. Changes in plasma AUC and CL_R of GCDCA-S upon administration of inhibitors with different inhibitory potencies (weak to strong). Different I/Ki ratios relative to (A) rifampicin or (B) probenecid.

The simulations followed the same study design as reported by Tatosian *et al.*[26] and Willemin *et al.*[17] For OATP1B3-mediated DDIs, AUC and CL_R were evaluated following a single dose of inhibitors. For the OAT3-mediated DDIs, AUC and CL_R were assessed at steady state after the inhibitors were administered every six hours. X-axis represents I/Ki ratios relative to rifampicin (0.001-1) or probenecid (0.01-1). Ratio of 1 corresponds rifampicin or probenecid. Left Y-axis and black symbols show the predicted GCDCA-S plasma AUC. Right Y-axis and white symbols represent the predicted GCDCA-S CL_R.

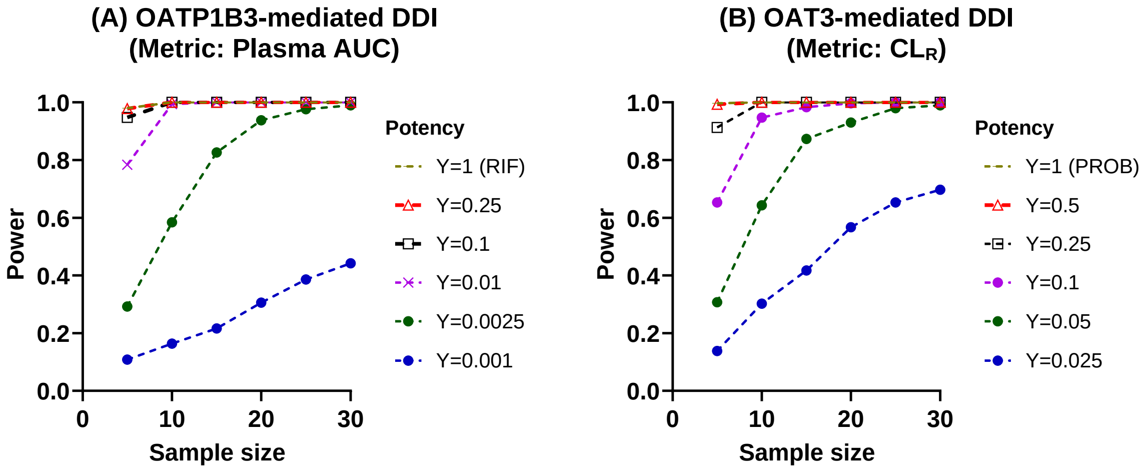

Figure S4. Power curves at significance levels (α=0.05) for the different hypothetical I/Ki ratios (Y) based on a one-sample paired t-test of the ratio of logarithmic transformed AUC or CL_R. (A) Power to detect OATP1B3-mediated DDIs based on changes in GCDCA-S plasma AUC, with I/Ki ratios ranging from 0.001 to 1 relative to that of rifampicin. (B) Power to detect OAT3-mediated DDIs based on changes in GCDCA-S CL_R, with I/Ki ratios ranging from 0.01 to 1 relative to that of probenecid. Ratios of 1 correspond to equivalent I/Ki ratio of rifampicin or probenecid, respectively.

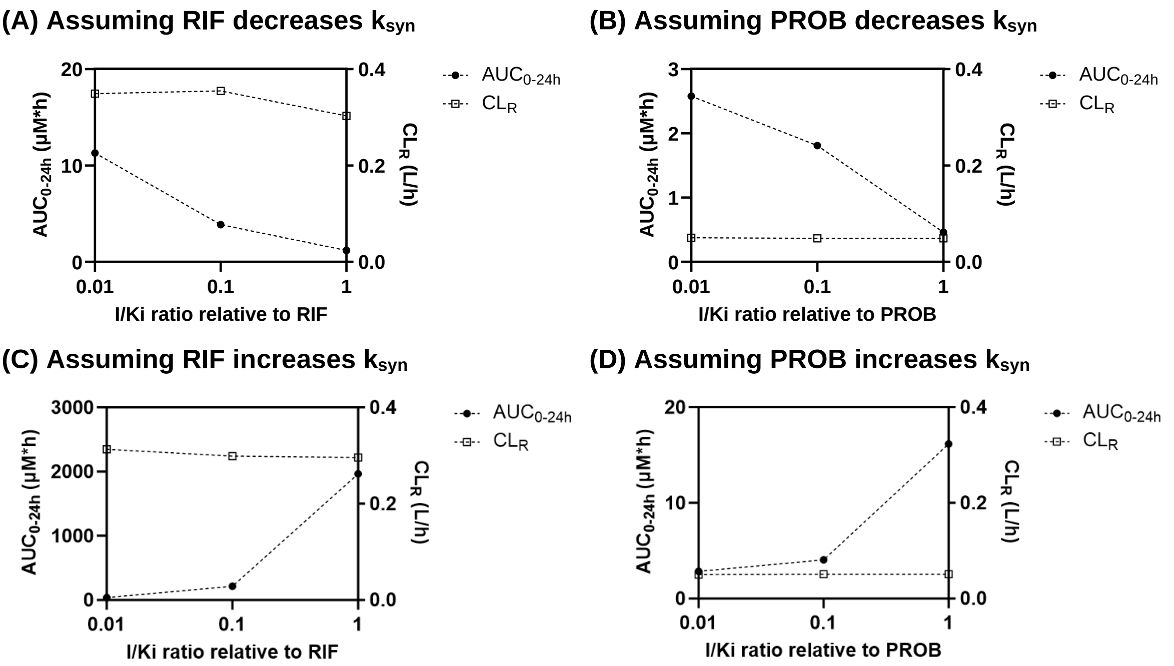

Figure S5. Changes in plasma AUC and CL_R of GCDCA-S assuming an additional effect of transporter inhibitors on GCDCA-S k_{syn}. (A) rifampicin decreases GCDCA-S k_{syn} (B) probenecid decreases k_{syn} (C) rifampicin increases k_{syn} (D) probenecid increases k_{syn}

The simulations followed the same study design as reported by Tatosian *et al.*[26] and Willemin *et al.*[17] For OATP1B3-mediated DDIs, AUC and CL_R were evaluated following a single dose of inhibitors. For the OAT3-mediated DDIs, AUC and CL_R were assessed at steady state after the inhibitors were administered every six hours. These simulations are based on the assumption of an immediate and direct increase or decrease of SULT2A1 activity (reflected as k_{syn} in POP-PK model). X-axis represents I/Ki ratios relative to rifampicin or probenecid. Left Y-axis and black symbols show the predicted GCDCA-S plasma AUC. Right Y-axis and white symbols represent the predicted GCDCA-S CL_R. Ratio of 0 corresponds to scenario of no effect of inhibitors on GCDCA-S k_{syn} (transporter inhibition only).

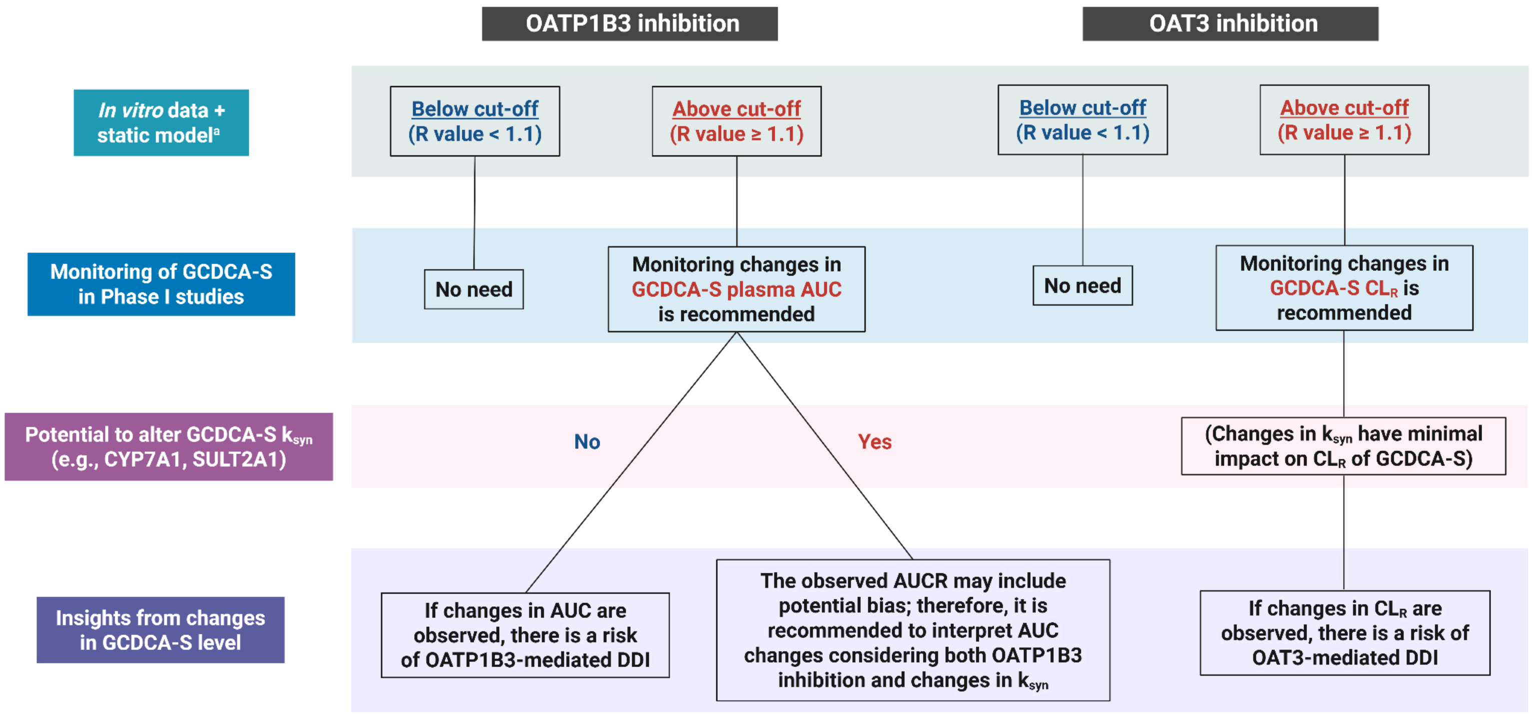

Figure S6. Proposed decision tree for leveraging GCDCA-S interaction data in plasma and urine to assess OATP1B3- and OAT3-mediated DDI risk in Phase I clinical trials.

At this stage, the framework serves as qualitative guidance and lacks definitive cut-off values to support the decision-making process. To establish such values, GCDCA-S data with OATP1B3/OAT3 inhibitors with varying potencies are necessary, as it has been accomplished for coproporphyrin-I.[27,28] [a] Cut-off values based on the current regulatory guidelines[29–31] This basic approach aims to minimize false-negative predictions, but are conversely associated with high false-positive rates, potentially leading to unnecessary follow-up clinical DDI studies.[32,33] AUC, area under the plasma concentration-time curve; CL_R, renal clearance; k_{syn}, synthesis rate; DDI, drug-drug interaction. This figure was created with BioRender.com.

REFERENCE

1. Orozco, C. C. *et al.* Characterization of Bile Acid Sulfate Conjugates as Substrates of Human Organic Anion Transporting Polypeptides. *Mol. Pharm.* **20**, 3020–3032 (2023).
2. Robbins, J. A. *et al.* Acute and Chronic Effects of Rifampin on Letermovir Suggest Transporter Inhibition and Induction Contribute to Letermovir Pharmacokinetics. *Clin. Pharmacol. Ther.* **111**, 664–675 (2022).
3. Takehara, I. *et al.* Comparative Study of the Dose-Dependence of OATP1B Inhibition by Rifampicin Using Probe Drugs and Endogenous Substrates in Healthy Volunteers. *Pharm. Res.* **35**, 138 (2018).
4. Mori, D. *et al.* Dose-Dependent Inhibition of OATP1B by Rifampicin in Healthy Volunteers: Comprehensive Evaluation of Candidate Biomarkers and OATP1B Probe Drugs. *Clin. Pharmacol. Ther.* **107**, 1004–1013 (2020).
5. Burra, P. *et al.* Hepatic immune regulation and sex disparities. *Nat. Rev. Gastroenterol. Hepatol.* **21**, 869–884 (2024).
6. Xie, G. *et al.* Profiling of Serum Bile Acids in a Healthy Chinese Population Using UPLC–MS/MS. *J. Proteome Res.* **14**, 850–859 (2015).
7. Grant, S. M. & DeMorrow, S. Bile Acid Signaling in Neurodegenerative and Neurological Disorders. *Int. J. Mol. Sci.* **21**, 5982 (2020).
8. Barnett, S. *et al.* Gaining Mechanistic Insight Into Coproporphyrin I as Endogenous Biomarker for OATP1B-Mediated Drug–Drug Interactions Using Population Pharmacokinetic Modeling and Simulation. *Clin. Pharmacol. Ther.* **104**, 564–574 (2018).
9. Ahmad, A. *et al.* Population pharmacokinetic modeling and simulation to support qualification of pyridoxic acid as endogenous biomarker of OAT1/3 renal transporters. *CPT Pharmacomet. Syst. Pharmacol.* **10**, 467–477 (2021).
10. Alnouti, Y. Bile Acid Sulfation: A Pathway of Bile Acid Elimination and Detoxification. *Toxicol. Sci.* **108**, 225–246 (2009).
11. Takehara, I., Watanabe, N., Mori, D., Ando, O. & Kusuhara, H. Effect of Rifampicin on the Plasma Concentrations of Bile Acid-*O*-Sulfates in Monkeys and Human Liver-Transplanted Chimeric Mice With or Without Bile Flow Diversion. *J. Pharm. Sci.* **108**, 2756–2764 (2019).

12. Cowen, A. R., Korman, M. G., Hofmann, A. F. & Cass, O. W. Metabolism of Lithocholate in Healthy Man: I. Biotransformation and biliary excretion of intravenously administered lithocholate, lithocholylglycine, and their sulfates. *Gastroenterology* **69**, 59–66 (1975).

13. Voronova, V. *et al.* A Physiology-Based Model of Bile Acid Distribution and Metabolism Under Healthy and Pathologic Conditions in Human Beings. *Cell. Mol. Gastroenterol. Hepatol.* **10**, 149 (2020).

14. Pattni, S. & Walters, J. R. F. Recent advances in the understanding of bile acid malabsorption. *Br. Med. Bull.* **92**, 79–93 (2009).

15. Molino, G., Hofmann, A. F., Cravetto, C., Belforte, G. & Bona, B. Simulation of the metabolism and enterohepatic circulation of endogenous chenodeoxycholic acid in man using a physiological pharmacokinetic model. *Eur. J. Clin. Invest.* **16**, 397–414 (1986).

16. Takehara, I. *et al.* Investigation of Glycochenodeoxycholate Sulfate and Chenodeoxycholate Glucuronide as Surrogate Endogenous Probes for Drug Interaction Studies of OATP1B1 and OATP1B3 in Healthy Japanese Volunteers. *Pharm. Res.* **34**, 1601–1614 (2017).

17. Willemin, M.-E. *et al.* Clinical Investigation on Endogenous Biomarkers to Predict Strong OAT-Mediated Drug–Drug Interactions. *Clin. Pharmacokinet.* **60**, 1187–1199 (2021).

18. Chan, G. H. *et al.* Evaluation of the Selectivity of Several Organic Anion Transporting Polypeptide 1B Biomarkers Using Relative Activity Factor Method. *Drug Metab. Dispos.* **51**, 1089–1104 (2023).

19. Yoshida, K., Guo, C. & Sane, R. Quantitative Prediction of OATP-Mediated Drug-Drug Interactions With Model-Based Analysis of Endogenous Biomarker Kinetics. *CPT Pharmacomet. Syst. Pharmacol.* **7**, 517–524 (2018).

20. Yoshikado, T. *et al.* PBPK Modeling of Coproporphyrin I as an Endogenous Biomarker for Drug Interactions Involving Inhibition of Hepatic OATP1B1 and OATP1B3. *CPT Pharmacomet. Syst. Pharmacol.* **7**, 739–747 (2018).

21. Takita, H. *et al.* PBPK Model of Coproporphyrin I: Evaluation of the Impact of SLCO1B1 Genotype, Ethnicity, and Sex on its Inter-Individual Variability. *CPT Pharmacomet. Syst. Pharmacol.* **10**, 137–147 (2021).

22. Kimoto, E. *et al.* Biomarker-Informed Model-Based Risk Assessment of Organic Anion Transporting Polypeptide 1B Mediated Drug-Drug Interactions. *Clin. Pharmacol. Ther.* **111**, 404–415 (2022).

23. Takita, H. *et al.* Coproporphyrin I as an Endogenous Biomarker to Detect Reduced OATP1B Activity and Shift in Elimination Route in Chronic Kidney Disease. *Clin. Pharmacol. Ther.* **112**, 615–626 (2022).

24. Ujihira, Y., Tan, S. P. F., Scotcher, D. & Galetin, A. Genotype, Ethnicity, and Drug–Drug Interaction Modeling as Means of Verifying Transporter Biomarker PBPK Model: The Coproporphyrin-I Story. *CPT Pharmacomet. Syst. Pharmacol.* doi:10.1002/psp4.70008

25. Tsuruya, Y. *et al.* Investigation of Endogenous Compounds Applicable to Drug–Drug Interaction Studies Involving the Renal Organic Anion Transporters, OAT1 and OAT3, in Humans. *Drug Metab. Dispos.* **44**, 1925–1933 (2016).

26. Tatosian, D. A. *et al.* A Microdose Cocktail to Evaluate Drug Interactions in Patients with Renal Impairment. *Clin. Pharmacol. Ther.* **109**, 403–415 (2021).

27. Galetin, A. *et al.* Membrane transporters in drug development and as determinants of precision medicine. *Nat. Rev. Drug Discov.* **23**, 255–280 (2024).

28. Kikuchi, R. *et al.* Utilization of OATP1B Biomarker Coproporphyrin-I to Guide Drug–Drug Interaction Risk Assessment: Evaluation by the Pharmaceutical Industry. *Clin. Pharmacol. Ther.* **114**, 1170–1183 (2023).

29. International Council for Harmonisation. ICH Harmonised Guideline - Drug Interaction Studies M12. <https://www.ema.europa.eu/en/documents/scientific-guideline/ich-m12-guideline-drug-interaction-studies-step-5_en.pdf> (2024).

30. Food and Drug Administration. In vitro drug interaction studies – Cytochrome P450 enzyme- and transporter-mediated drug interactions guidance for industry <https://www.fda.gov/regulatory-information/search-fda-guidance-documents/in-vitro-drug-interaction-studies-cytochrome-p450-enzyme-and-transporter-mediated-drug-interactions> (2020).

31. European Medicines Agency. Guideline on the investigation of drug interactions (CPMP/EWP/560/95/Rev. 1 Corr. 2**) <https://www.ema.europa.eu/en/documents/scientific-guideline/guideline-investigation-drug-interactions-revision-1_en.pdf> (2012).

32. Vaidyanathan, J., Yoshida, K., Arya, V. & Zhang, L. Comparing Various In Vitro Prediction Criteria to Assess the Potential of a New Molecular Entity to Inhibit Organic Anion Transporting Polypeptide 1B1. *J. Clin. Pharmacol.* **56**, S59–S72 (2016).

33. Yoshida, K. *et al.* In Vitro–In Vivo Extrapolation of Metabolism- and Transporter-Mediated Drug–Drug Interactions—Overview of Basic Prediction Methods. *J. Pharm. Sci.* **106**, 2209–2213 (2017).
